# Plant-Derived Modifiers for Antimicrobial Soft Denture Liners: A Review

**DOI:** 10.3390/ijms262210848

**Published:** 2025-11-08

**Authors:** Patrycja Kula, Grzegorz Chladek, Izabela Barszczewska-Rybarek

**Affiliations:** 1 Department of Physical Chemistry and Technology of Polymers, Faculty of Chemistry, Silesian University of Technology, Strzody 9 Str., 44-100 Gliwice, Poland; patrycja.kula@polsl.pl; 2 Materials Research Laboratory, Faculty of Mechanical Engineering, Silesian University of Technology, Konarskiego 18A Str., 44-100 Gliwice, Poland; grzegorz.chladek@polsl.pl

**Keywords:** soft lining materials, *Candida albicans*, antifungal activity, natural bioactive compounds, phytotherapeutics

## Abstract

This review examines strategies to enhance the antifungal properties of commercial soft lining materials (SLMs) through modification with plant-derived oils, extracts, and powders. These natural bioactive compounds act via multiple mechanisms, including disruption of fungal cell membranes, inhibition of biofilm formation, and interference with *Candida albicans* metabolism, the pathogen causing denture-associated candidiasis. Their incorporation into SLM provides localized antifungal activity at the denture–mucosa interface. The review highlights *Aloe vera* (aloe), *Azadirachta indica* (neem), *Ocimum basilicum (basil)*, *Melaleuca alternifolia* (tea tree), *Cocos nucifera* (coconut), *Allium sativum* (garlic), *Thymus vulgaris* (thyme), and chitosan as notable sources of phytotherapeutics that consistently inhibit *C. albicans* growth. In addition to antimicrobial effects, studies assessed both intrinsic (hardness, tensile strength, tear strength) and interfacial (bond strength) mechanical properties, as well as surface roughness. Most formulations maintained acceptable mechanical performance and improved surface smoothness. Key limitations include rapid leaching of active compounds, variability in testing methods, and insufficient in vivo and cytotoxicity data. Future research should prioritize the high-quality purification of natural extracts, the isolation of well-defined bioactive compounds, and the design of systems enabling selective and sustained release of these agents, ensuring reproducibility, enhanced stability, and clinical reliability of next-generation bioactive SLMs.

## 1. Introduction

**Figure 1:**
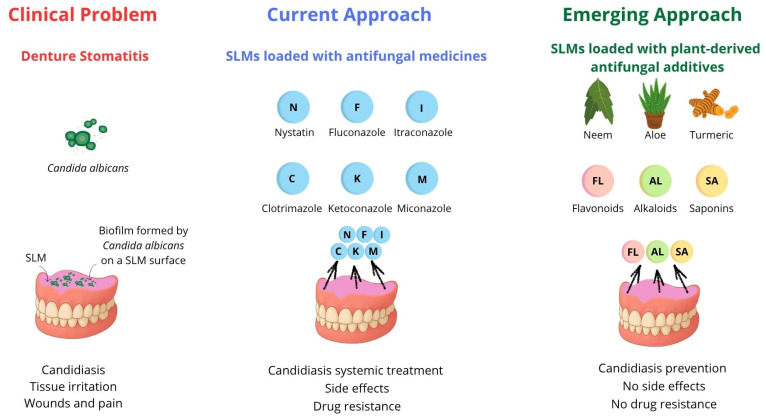
Denture stomatitis management with SLMs modified with drugs and plant-derived additives.

Denture-related infections remain a significant challenge in prosthetic dentistry. Soft lining materials (SLMs), commonly used to enhance denture fit and comfort, do not inhibit the growth of pathogenic microorganisms, contributing to a high incidence of denture stomatitis, predominantly caused by *Candida albicans* [1,2,3]. Candidiasis frequently affects denture wearers with poor oral hygiene, prolonged denture use, unhealthy diet, smoking habits, micro-injuries, reduced salivary flow, hormonal imbalances, or compromised immunity. High-risk groups include older adults, diabetics, chemotherapy patients, and individuals with HIV [4,5,6,7]. Over the past two decades, its prevalence has risen significantly, now affecting even healthy individuals [8]. Conventional approaches to managing this condition rely on incorporating antifungal medicines, such as nystatin, clotrimazole, miconazole, fluconazole, and itraconazole, into SLMs [2]. However, the limitations of conventional antifungal therapeutics, including systemic side effects and emerging drug resistance [9], have prompted interest in plant-derived bioactive compounds. These natural additives offer broad-spectrum antimicrobial activity with lower cytotoxicity and minimal side effects. Phytochemicals such as flavonoids, terpenes, saponins, and alkaloids have demonstrated promising antifungal efficacy and may serve as safer, effective alternatives for modifying SLMs (Figure 1).

Unlike previous reviews, which focused on the incorporation of synthetic antifungal medicines into SLMs, this review offers a comprehensive and comparative analysis of current advancements in the development of SLMs modified with plant-derived additives. This is the first literature review on this topic so far. It highlights the influence of plant-derived additives on antifungal efficacy, antibacterial efficiency, biocompatibility, mechanical properties and roughness of commercial SLMs. By emphasizing the unique benefits of plant-derived additives, this article aims to establish their role as a viable and sustainable solution in the research of modern prosthodontic biomaterials.

## 2. Classification of SLMs

SLMs are classified based on service time into temporary soft lining materials (T-SLMs) and long-term soft lining materials (LT-SLMs) [2].

T-SLMs include tissue conditioners (TCs) and short-term soft lining materials (ST-SLMs), both representing acrylic-based SLMs (A-SLMs), typically comprising two components: a powder and a liquid. TCs are classified as self-curing systems. The gelation, occurring at oral cavity temperature, involves the plasticization of poly(ethyl methacrylate) (PEMA) or poly(methyl methacrylate) (PMMA) with the plasticizer-solvent system. In contrast, ST-SLMs contain an initiating system and have a more complex composition of a liquid component, being a mixture of a plasticizer, a solvent (commonly ethanol), and methacrylate monomers such as methyl methacrylate (MMA), ethyl methacrylate (EMA), n-butyl methacrylate (BuMA), ethylene glycol dimethacrylate (EGDMA), and triethylene glycol dimethacrylate (TEGDMA). Additionally, an accelerator for initiating free radical polymerization, typically N,N-dimethyl-*p*-toluidine (DMPT), is included. The initiator for the free radical polymerization, usually benzoyl peroxide (BPO), is mixed into the powder. ST-SLMs are heat-curing systems, which undergo gelation at elevated temperature through the simultaneous plasticization of PEMA (alternatively PMMA) and the polymerization of the methacrylate monomers, forming a crosslinked structure when dimethacrylates are present, thereby extending service time. LT-SLMs usually are crosslinked silicone elastomers (S-SLMs) available as one- or two-component systems. Single-component S-SLMs consist of vinyl-terminated polydimethylsiloxanes and organic peroxide (typically BPO). Their polymerization requires high temperatures. In contrast, two-component S-SLMs consist of a catalyst paste that combines vinyl-terminated polydimethylsiloxanes with a platinum catalyst, and a base paste made of vinyl-terminated and hydride-terminated polydimethylsiloxanes. The reaction between the catalyst and the base occurs at room temperature [2].

## 3. The Concept of Antimicrobial SLMs with Plant-Derived Additives

**Figure 2:**
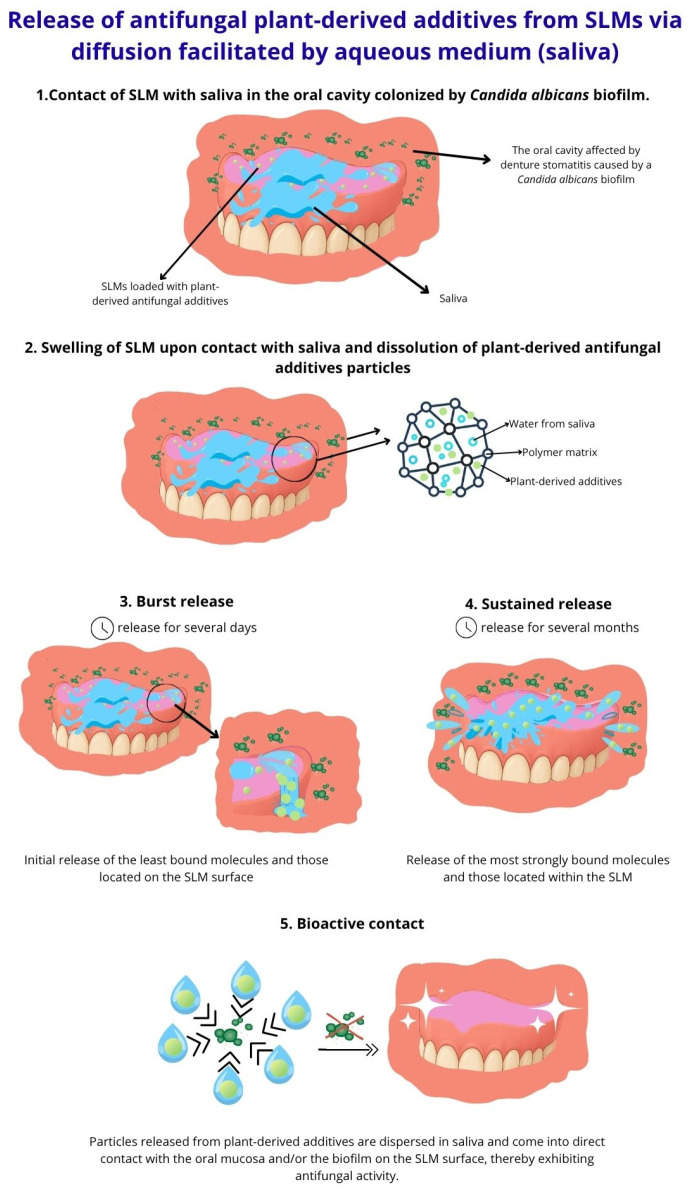
Release of antifungal plant-derived additives from SLM to saliva.

The concept of antimicrobial SLMs with plant-derived additives is based on their ability to release bioactive compounds into saliva, similar to controlled drug-release systems, allowing direct action on the oral soft tissues without toxic effects (Figure 2). To prevent denture-related candidiasis, these additives must primarily exhibit antifungal activity against *C. albicans*.

A wide variety of plant-derived compounds exhibit potent antifungal activity against *C. albicans* through diverse bioactive compounds and mechanisms. *Aloe vera* (aloe) [10,11], *Azadirachta indica* (neem) [12,13], and Triphala (three fruits) [14,15] contain anthraquinones, alkaloids, tannins, and flavonoids that disrupt fungal membranes, inhibit biofilm formation, and suppress hyphal growth. *Origanum vulgare* (oregano) [16,17], *Thymus vulgaris* (thyme) [18], and *Ocimum* species (basil, tulsi) [19,20] owe their activity mainly to terpenes and phenolics such as thymol, carvacrol, and eugenol, which damage cell membranes and inhibit ergosterol synthesis. The main bioactive compound in *Cocos nucifera* (coconut), lauric acid, exerts antifungal effects by disrupting the fungal cell membrane and causing leakage of cellular contents [21,22]. The major bioactive component in *Allium sativum* (garlic) is allicin, which damages the cell wall, inhibiting key enzymes and disrupting metabolism, leading to cell lysis [23,24]. *Melaleuca alternifolia* (tea tree) and *Cymbopogon citratus* (lemongrass) oils, rich in terpenoids like terpinen-4-ol and citral, disrupt membrane integrity and induce oxidative stress [25,26]. *Curcuma longa* (turmeric), *Zingiber officinale* (ginger), and *Cinnamomum* (cinnamon) contain polyphenols (curcumin, gingerol, cinnamaldehyde) that interfere with fungal signaling, enzyme activity, and cell wall synthesis [27,28,29]. *Nigella sativa* (black seed) and *Linum usitatissimum* (flaxseed) alter ergosterol and lipid metabolism [30,31], while *Centratherum anthelminticum* (black cumin) and *Litsea cubeba* show similar effects via terpenoids [32,33]. *Piper betle* demonstrates antifungal action through membrane disruption and inhibition of ergosterol biosynthesis [34,35]. Recently, Rana et al. demonstrated that anticancer flavonoids such as prunin and isorhamnetin also exhibit notable antifungal activity. These compounds disrupt fungal cell membranes and induce oxidative stress, positioning them as mechanistic analogues to other antifungal flavonoids [36,37]. Finally, chitosan, a natural polymer, exhibits antifungal activity through electrostatic interactions with the fungal cell surface [38,39].

## 4. Methodology

This narrative systematic literature review combines a systematic methodology with a narrative synthesis approach.

The literature regarding the main topic, i.e., SLMs modified with plant-derived additives, was systematically reviewed in accordance with the PRISMA guidelines. A comprehensive literature search was conducted across four scientific databases: Scopus, Lens, Web of Science, and PubMed, covering the period from January 2015 to September 2025. Search queries were constructed using combinations of keywords and Boolean operators (AND, OR) associated with the review’s main concepts. The principal search terms included: “soft liner”, “antifungal”, “mechanical”, “roughness”, “inorganic”, “nanoparticles”, “drug”, “antibiotic”, and “medicine.” To ensure completeness, manual searches were also conducted to identify additional relevant publications not indexed in the selected databases.

Studies were included if they met the following criteria: (i) published in peer-reviewed journals between 2015 and 2025, (ii) written in English, (iii) directly addressed the core topic and objectives of this review, (iv) contained experimental data, systematic analyses, or comprehensive discussions relevant to antimicrobial SLMs modified with plant-derived compounds.

Studies were excluded if they: (i) were conference abstracts, editorials, or book chapters, (ii) lacked sufficient methodological or analytical detail, (iii) were duplicates or unavailable in full text, (iv) focused on modifications involving inorganic additives or medicines, pharmaceutics, or antiseptics.

**Figure 3:**
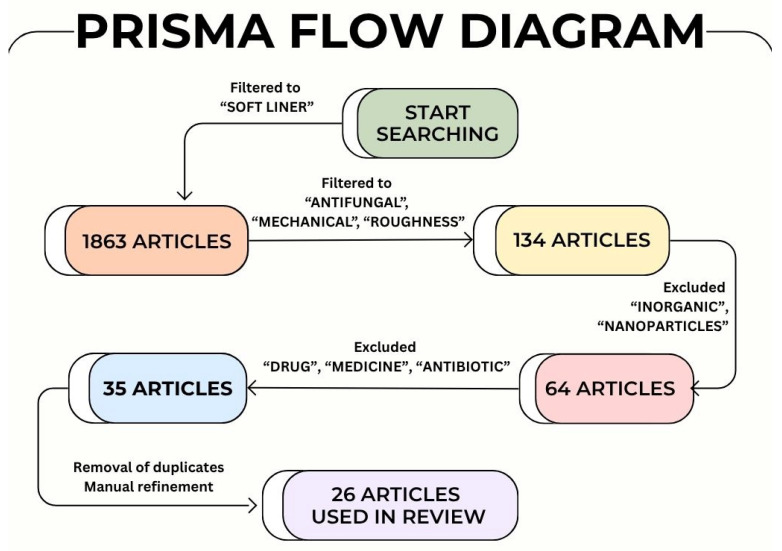
Flowchart of the review methodology on SLMs modified with plant-derived additives.

Figure 3 illustrates the selection process and keyword co-occurrence flowchart, summarizing the identification and screening stages and highlighting research clusters on SLMs modified with plant-derived antifungals in current literature.

The initial database search yielded 1863 records. Following keyword refinement using “soft liner” AND “antifungal” AND “mechanical” AND “roughness”, and excluding “inorganic”, “nanoparticles”, “drugs”, “medicine”, and “antibiotics”, 35 potentially relevant articles were identified. Additionally, 8 supplementary records were retrieved through manual searches and cross-referencing. After the removal of 10 duplicate records, the remaining 33 full-text articles were screened for eligibility. Following manual refinement by limiting the studies to findings strictly related to SLMs modified with plant-derived additives, a total of 26 studies were ultimately included in the final review (The Excel file containing these articles can be found in the Supplementary Materials). The data from these selected studies were then systematically organized according to major research themes, including plant-derived extracts, essential oils, herbal formulations, SLMs type, methods of modification, in vitro and in vivo antifungal assays, in vitro antibacterial assays, biocompatibility assessment, mechanical properties, roughness, limitations and future perspectives. Each study was carefully reviewed to identify recurring trends, experimental outcomes, knowledge gaps, and limitations to outline emerging challenges and prospective directions for future research on SLMs modified with plant-derived antimicrobial additives.

In addition to the directly identified studies on SLMs modified with plant-derived antifungals, a further 130 publications were manually searched and critically reviewed. This broader literature base supported and contextualized the findings, and allowed for a critical assessment of trends, gaps, and inconsistencies in the field.

## 5. Discussion

The reviewed studies indicate that plant-derived additives can enhance the antifungal potential of SLMs. However, this effect varies considerably depending on the plant species, the bioactive form used, the method of incorporation, and the type and composition of the SLM.

**Figure 4:**
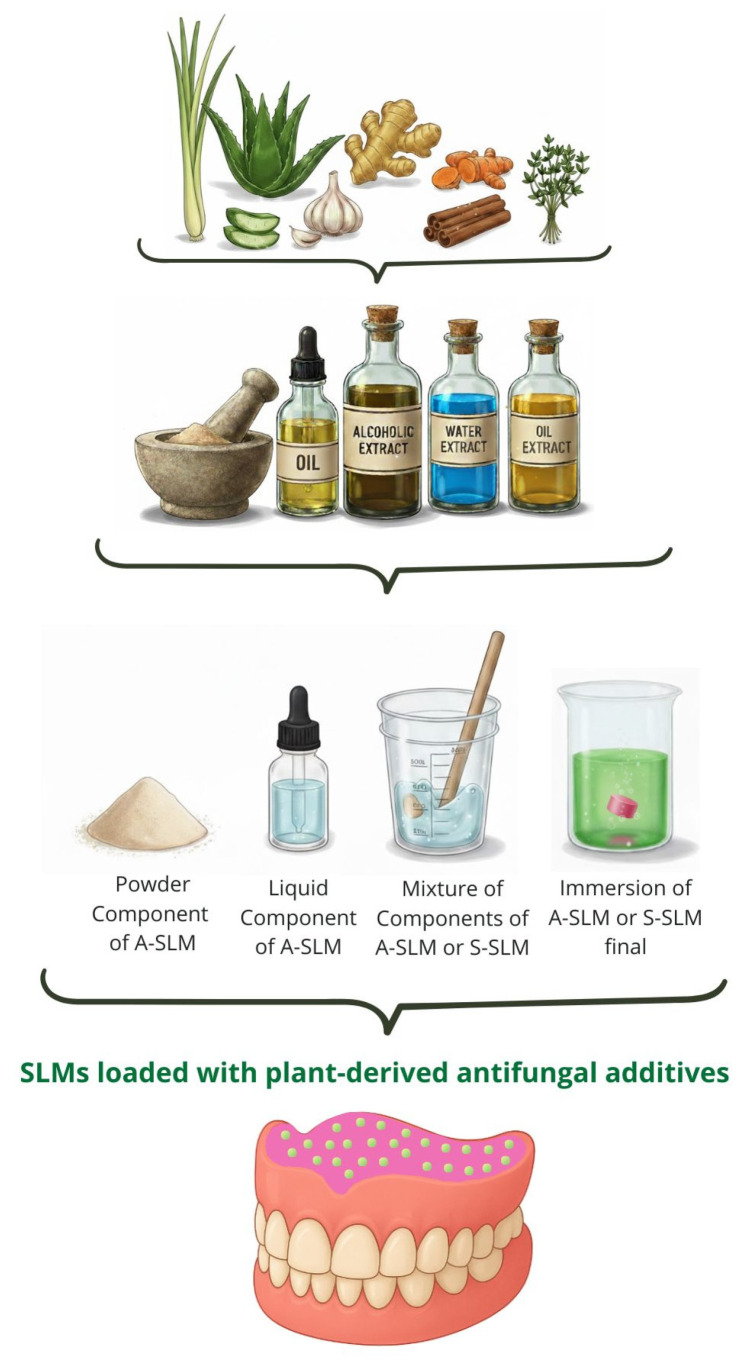
Forms and methods of incorporating plant-derived additives into SLMs.

The plant-derived additives used for SLM modification included essential oils, solutions, and liquid or dry extracts. These bioactive substances were incorporated into the material by adding them to the powder component, the liquid component, or a mixture of both immediately before polymerization. Alternatively, a post-fabrication approach was employed in which ready-to-use SLMs were immersed in phytochemical solutions, extracts, or oils to impart antifungal properties (Figure 4).

Table 1 provides a comparative overview of acrylic-based SLMs, including TCs and ST-SLMs, modified with plant-derived compounds via addition to the powder, liquid, or powder–liquid mixture of the SLM. The table is organized according to the plant species and family, plant-derived additive content and form (powder, oil, or solution), the SLM type, trade name, manufacturer, information on processing, component enriched, and the corresponding in vitro antimicrobial and mechanical properties tested.

Table 2 provides a comparative overview of silicone-based SLMs (LT-SLMs) modified with plant-derived compounds via addition to the powder–liquid mixture of the SLM. The table is organized according to the plant species and family, plant-derived additive content and form (powder, oil, or solution), the SLM type, trade name, manufacturer, information on processing, component enriched, and the corresponding in vitro antimicrobial and mechanical properties tested.

Table 3 provides a comparative overview of tissue conditioners (TCs) modified with plant-derived compounds via immersion method. The table is organized according to the plant species and family, immersion liquid (volume), the SLM type, trade name, manufacturer, information on processing, and the corresponding in vitro antimicrobial and mechanical properties tested.

While Table 1, Table 2 and Table 3 summarize the antifungal performance of SLMs according to material type, plant species, and method of incorporation, it should be noted that multiple sources of methodological bias may influence the analysis.

The following general issues in methodological variability and potential bias can be distinguished:

- SLM type (TC, ST-SLM, LT-SLM).
- SLM composition, including polymer type (A-SLM, S-SLM), plasticizer, and ethanol content.
- Differences in extraction methods for bioactive compounds.
- Variations in the form of bioactive compounds (essential oil, powder, or dried plant parts).
- Additive incorporation method, such as direct admixing, immersion, or nanoparticle dispersion.
- Additive-enriched component in direct admixing (added to liquid, powder, or pre-formulated mixture).
- Inconsistent reporting of additive concentration, rarely expressed as weight or volume fractions relative to total sample mass. Often, fixed amounts were added to liquid or powder according to manufacturer-recommended ratios, preventing standardized comparison.
- Additive concentration range tested.
- Immersion medium, e.g., pure additive oil or solvent.

This structure highlights the multiple sources of variability that can influence the reproducibility and comparability of results in studies on plant-modified SLMs.

Despite this variability, a stepwise analysis of the available studies enabled the identification of important trends and facilitated a discussion of the results, as presented in the following sections.

### 5.1. In Vitro Antifungal Efficacy

The following section discusses the in vitro antifungal properties of SLMs modified with plant-derived additives against *C. albicans*, highlighting trends associated with plant species, incorporation method, and SLM type (Table 4). Nineteen plant-derived species and compounds were identified. The most frequently tested were *M. alternifolia* (6 times), *A. indica* (4 times), and chitosan (3 times). Others, including *A. vera*, *A. sativum*, *O. vulgare*, *T. vulgaris*, *O. basilicum*, *O. sanctum*, *C. nucifera*, *Cinnamomum sp.*, *C. longa*, *Z. officinale*, *N. sativa*, *L. cubeba*, *C. citratus*, *L. usitatissimum*, *C. anthelminticum*, and *P. betle*, were each evaluated once.

Songsang et al. [53] investigated *L. cubeba* oil (5–30 vol./vol.%) in three TCs (A-SLMs, Table 1 and Table 4): GC Soft Liner, Coe-Comfort, and Visco-gel. No activity was observed at 5 vol./vol.%, whereas concentrations of 10 vol./vol.% and higher produced increasing inhibition zones. IZD was also dependent on the TC and decreased followingly: Visco-gel > Coe-Comfort > GC Soft Liner, with IZDs at 10, 20, and 30 vol./vol.% of 12.09, 16.56, 24.68 mm (Visco-gel); 10.22, 14.07, 22.61 mm (Coe-Comfort); and 7.61, 13.19, 22.42 mm (GC Soft Liner), all exceeding the nystatin control (12 mm). This highlighted both the antifungal potential of *L. cubeba* oil and the influence of SLM composition, particularly the plasticizer type. The highest antifungal activity of Visco-gel can be explained by synergistic interaction between the triethyl citrate plasticizer and ethanol. Nikawa et al. [64] reported that ethanol content in SLM can significantly affect antifungal activity depending on the plasticizer, showing a positive correlation with benzyl salicylate but an inverse correlation with benzyl n-butyl phthalate. Supporting this, Truhlar et al. [65] observed that nystatin incorporated into Visco-gel exhibited greater antifungal activity than in Lynal, indicating that material composition itself influences antifungal performance. This highlights that both plasticizer type and its interaction with ethanol modulates antifungal efficacy. Benzyl benzoate plasticizer possesses intrinsic antifungal properties, contributing to intermediate activity of Coe-Comfort, which is the opposite of inert butyl phthalyl butyl glycolate, explaining the weakest activity of GC Soft Liner [66].

Vankadara et al. [50] consistently found higher antifungal activity of Visco-gel than GC Soft Liner due to compositional differences. They investigated *M. alternifolia* oil (0.5–2.0 mL; 10–40 vol./vol.%) in these two TCs (A-SLM, TC, Table 1 and Table 4), observing a clear dose-dependent response for both. For Visco-gel, the IZDs increased from 19.0 mm (0.5 mL) to 20.8 mm (2 mL) at 10 vol./vol.% and from 21.6 mm (1.5 mL) to 26.4 mm (2 mL) at 40 vol./vol.%. The maximum inhibition (26.4 mm at 2 mL, 40 vol./vol.%) slightly exceeded the nystatin control (22.6 mm). In contrast, GC Soft Liner exhibited weaker activity at each condition, with IZDs increased from no inhibition zone observed (0.5 mL) to 17.4 mm (2 mL) at 10 vol./vol.% and from 15.2 mm (0.5 mL) to 23.2 mm (2 mL) at 40 vol./vol.%. The latter was comparable to the control. These results were supported by CFU analysis. For Visco-gel, *C. albicans* colonization decreased from 4.0 ×10^5^ CFU/mL (0.5 mL) to 2.24 ×10^5^ CFU/mL (2 mL) at 10 vol./vol.%, and from 1.72 ×10^5^ to 0.88 ×10^5^ CFU/mL at 40 vol./vol.%. For GC Soft Liner, the corresponding reductions were from 4.88 ×10^5^ to 2.86 ×10^5^ CFU/mL (10 vol./vol.%) and from 3.8 ×10^5^ to 1.08 ×10^5^ CFU/mL (40 vol./vol.%). Overall, Visco-gel consistently exhibited larger IZDs and lower colonization than GC Soft Liner at corresponding doses, confirming that matrix composition strongly affects antifungal performance. However, it is worth noting that highly effective *C. albicans* reduction was achieved for both TCs, which reached 88% for Visco-gel and 86% for GC Soft Liner (for 2 mL, 40 vol./vol.%).

Pachava et al. [60] confirmed the notable antifungal activity of *M. alternifolia* oil (15 wt.%) in the silicone-based GC Reline Extra Soft (S-SLM, LT-SLM, Table 2 and Table 4). The modified SLM exhibited markedly lower log CFU/mm^2^ values (2.1, 2.8, and 3.1 at 1, 30, and 60 days, respectively) compared to the unmodified control (7.1, 6.5, and 6.8), corresponding to fungal growth reductions of 70.4%, 56.9%, and 54.4%. Although antifungal activity declined gradually over time, the activity against *C. albicans* remained evident after 60 days, indicating the strong and sustained antifungal activity of S-SLMs.

On the contrary, Patil et al. [63] reported only limited antifungal activity when incorporating *M. alternifolia* into GC Soft Liner (A-SLM, TC, Table 1 and Table 4) using an immersion method (50 vol./vol.% DMSO solution). This result indicates that the antifungal efficacy of *M. alternifolia* depends strongly on its concentration, incorporation method, and the TC. Consequently, the immersion method appears least effective, and using undiluted or higher-concentration oil formulations with more compatible TCs could be recommended to achieve stronger SLM antifungal performance. In the same study, *M. alternifolia* showed lower antifungal activity than *C. citratus* (MIC 0.03125 vol./vol.%, 50% inhibition) and *T. vulgaris* (MIC 0.0625 vol./vol.%, 30% inhibition), with *M. alternifolia* exhibiting only minimal biofilm reduction at 0.25 vol./vol.%. This is consistent with the high citral (*C. citratus*) and thymol, and carvacol (*T. vulgaris*) content. The lowest antifungal activity of *M. alternifolia* oil can be attributed to the high volatility of terpinen-4-ol, which probably reduces potency over time, particularly during extended incubation.

AlHamdan et al. [62] compared *M. alternifolia* and *A. indica* extracts incorporated into GC Soft Liner (A-SLM, TC, Table 3 and Table 4) by immersion. *A. indica* achieved slightly higher antifungal efficacy (log CFU/mL = 2.15) than *M. alternifolia* (2.31), both acting comparably to the chlorhexidine control (2.36). These close values suggest that both extracts impart similar fungicidal effects, with *A. indica* demonstrating slightly stronger activity, which can be attributed to its more hydrophobic bioactive components (azadirachtin, nimbidin, gedunin) [12,13]. In contrast, the comparatively weaker activity of *M. alternifolia* may be attributed to the high volatility of terpinen-4-ol, which diminishes in potency over time [67]. The authors concluded that undiluted immersion can be effective if extract composition is compatible with the liner and the bioactive component has sufficient stability.

Singhania et al. [61] further showed that the combination of *M. alternifolia* with *A. indica* can produce a synergistic antifungal effect. Using the immersion technique with GC Soft Liner (A-SLM, TC, Table 3 and Table 4), *M. alternifolia* oil alone reduced fungal growth to a log CFU/mL of 2.30, whereas the combination of *M. alternifolia* with *A. indica* resulted in a near-complete inhibition, with a log CFU/mL of 0.40.

Kumar et al. [45] extended the comparative analysis beyond previous studies by evaluating the effects of *A. indica*, *M. alternifolia*, and *C. nucifera* oils (5–40 vol./vol.%) on the antifungal properties of Visco-gel (A-SLM, TC, Table 1 and Table 4). At 5 vol./vol.%, all plants exhibited low but measurable antifungal activity. The IZDs measured after 48 h were 6.35 mm for *A. indica* and 5.0 mm for both *M. alternifolia* and *C. nucifera*. At the highest tested concentration, 40 vol./vol.%, all materials achieved their maximum IZDs, with *A. indica* showing 20.10 mm, *C. nucifera* 16.55 mm, and *M. alternifolia* 15.35 mm. After 7 days, the same concentration-dependent trend persisted but with slightly reduced IZDs, indicating decreased long-term activity. At 40 vol./vol.%, *A. indica* maintained the highest inhibition (19.15 mm), followed by *C. nucifera* (16.20 mm) and *M. alternifolia* (14.75 mm). Regarding the optimum concentrations, these were determined based on the most pronounced increase in IZDs. For *A. indica*, the most significant improvement was observed at 15 vol./vol.%, where the IZD reached 12.20 mm after 48 h and remained high at 11.35 mm after 7 days. *C. nucifera* exhibited optimum at 20 vol./vol.%, with an IZD of 11.0 mm after 48 h and 9.70 mm after 7 days. *M. alternifolia* showed its most effective concentration at 25 vol./vol.%, achieving an IZD of 8.8 mm after 48 h and 8.0 mm after 7 days. These results indicate that all plant-derived oils maintained antifungal activity over time, though with reductions after prolonged incubation. The observed decreasing trend reflects the influence of oil composition and antifungal mechanism. The strongest performance of *A. indica* results from the richness of bioactive compounds such as azadirachtin, nimbidin, and gedunin [12,13]. *C. nucifera* showing moderate activity, is composed of medium-chain fatty acids [21,22]. Finally, *M. alternifolia*, appearing to be the weakest fungicide, contains highly volatile terpinen-4-ol [67,68].

In another study, Kumar et al. [44] confirmed complete inhibition of *C. albicans* with *A. indica* powder (50–500 µg) incorporated into GC Soft Liner (A-SLM, TC, Table 1 and Table 4), which persisted through 7 days. In the same study, *A. sativum* powder demonstrated significantly weaker activity, with complete inhibition persisting through 2 days. The lower antifungal efficacy of *A. sativum* can be attributed to allicin instability [69].

Mohammed et al. [47] compared *A. sativum*, *O. vulgare*, *N. sativa*, and *Z. officinale* (50 µg/mL) in Acrosoft (A-SLM, TC, Table 1 and Table 4), observing the following order of decreasing activity: *A. sativum* (IZD = 8.00 mm) > *O. vulgare* (IZD = 7.75 mm) > *Z. officinale* (negligible effect) ≈ *N. sativa* (no inhibition). These findings showed that less hydrophobic compounds, such as allicin (*A. sativum,* water solubility 24 g/L at 10 °C [70]), exhibit high activity due to facilitated diffusion into the aqueous oral environment. In contrast, poorly water-soluble compounds, such as thymoquinone (*N. sativa*, water solubility 0.1 g/100 mL (20 °C) [71]) and gingerol (*Z. officinale*, water solubility < 0.0001 mole fraction at < 130 °C [72]), showed negligible activity, likely due to limited release. Interestingly, *O. vulgare* exhibited strong antifungal activity despite its predominantly hydrophobic constituents, thymol and carvacrol. This effectiveness can be attributed to their synergistic interaction, which enhances membrane disruption and antifungal potency even at low concentrations [73,74].

Regarding antifungal mechanisms of the aforementioned bioactive compounds, one can conclude that hydrophilic enzyme-targeting compounds (e.g., allicin) typically show stronger activity in SLMs due to improved diffusion through the polymer matrix, while certain combinations of phenolics (e.g., thymol and carvacrol) maintain potency through membrane-disruptive synergy [73].

Consistent with this, Baygar et al. [59] observed a high sensitivity of *C. albicans* cells to the silicone-based Ufi Gel P (S-SLM, LT-SLM, Table 2) modified with carvacrol (10 mL), the principal monoterpenoid in *O. vulgare* oil. A notable IZD of 38.33 mm and a 98% biofilm reduction were recorded for the modified SLM.

Analysis of results for S-SLM: Ufi Gel P [59] and GC Reline Extra Soft [60] leads to the conclusion that silicone-based SLMs provide stronger inhibition than acrylic ones. It can be explained by the silicone hydrophobic character, which minimizes interactions with antifungal compounds, promoting their diffusion and release.

Muttagi et al. [46] evaluated *C. anthelminticum*, *O. sanctum*, and *L. usitatissimum* (600–1000 µL) in Visco-gel (A-SLM, TC, Table 1 and Table 4). Among the tested formulations, *C. anthelminticum* exhibited the strongest and most durable antifungal effect, with complete inhibition of fungal growth at 600–800 µL during the first 48 h, and for 800 µL even up to 72 h. After 72 h, the IZD decreased in a dose-dependent trend (800 µL—complete inhibition; 700 µL—49.33 mm; 600 µL—46.00 mm). By day 7, IZDs dropped to 31.66, 21.67, and 18.33 mm, respectively, indicating that higher oil content prolonged both intensity and duration of activity. *O. sanctum*-modified SLMs showed moderate antifungal activity. Complete inhibition was noted during the first 48 h for all concentrations except 600 µL (IZD = 40 mm). After 72 h, inhibition persisted for 600 µL (38.33 mm) and 700 µL (43.66 mm), with complete inhibition maintained only at 800 µL. By day 7, a notable reduction was observed (21.00, 28.00, and 29.66 mm for 600, 700, and 800 µL, respectively). In contrast, *L. usitatissimum* oil demonstrated only transient antifungal activity. Inhibition zones were observed for higher concentrations (700–1000 µL) at 24 h (20.33–21.00 mm), decreased slightly at 48 h (15.00–17.00 mm), and disappeared by 72 h, matching the control. Increasing concentrations beyond 800 µL did not enhance inhibition. These results show the following order of decreasing fungicidal activity: *C. anthelminticum* > *O. sanctum* > *L. usitatissimum*, with overall performance showing both concentration- and time-dependent behavior [46]. The strongest and most sustained activity of *C. anthelminticum* can be explained by the high diversity of its bioactive compounds, including sterols, terpenes, tannins, fatty acids, lactones, phenolics, flavonoids, cyclic polyols, and benzoic acid. Among these, terpenes are primarily hydrophobic, sterols are amphiphilic, and tannins possess both hydrophilic and hydrophobic regions, allowing multiple mechanisms of action [32]. *O. sanctum* exhibited moderate activity due to eugenol and other phenolics, which are generally hydrophobic and interfere with ergosterol synthesis [19]. In contrast, *L. usitatissimum* showed weak and short-lived antifungal effects, likely due to its chemical composition dominated by hydrophobic fatty acids, in the combination of the lack of strong membrane-disruptive or enzyme-targeting antifungal properties [30].

Muttagi et al. [46] supported their findings with a detailed analysis of mass reduction, as a parameter, which is closely related to the controlled release of bioactive compounds from SLMs into the oral environment [75]. The release rate determines the SLM antifungal performance, with rapid initial release producing strong initial activity and slower diffusion providing prolonged effectiveness [76]. Mass-loss analysis revealed faster initial release (24 h) for *O. sanctum* (6.7%) and slower, sustained release for *C. anthelminticum* (4.0%). After this stage, *C. anthelminticum*-modified samples reached a plateau, suggesting stabilization of the polymer–fungicide system and minimal further leaching. In contrast, *O. sanctum*-modified specimens showed a modest increase by 72 h (3.2%), likely due to water absorption. Comparison with antifungal activity indicates that mass reduction within the first 24 h is not directly correlated with antifungal efficacy. *C. anthelminticum* exhibited the strongest and most sustained antifungal effect, even though its mass loss was smaller than that of *O. sanctum*. The observed plateau in *C. anthelminticum* likely reflects its slower diffusion and controlled fungicidal release, associated with its diverse composition of bioactive compounds, leading to greater stability and prolonged activity. Water contact angle (WCA) measurements supported these findings, showing greater hydrophobicity and lower water uptake in *C. anthelminticum*-modified specimens.

Poolkerd et al. [34] investigated the antifungal activity of *P. betle* incorporated into GC Soft Liner (A-SLM, TC, Table 1 and Table 4), comparing extract and essential oil forms. Growth inhibition zones were first observed at 5 wt./wt.% for the extract and 20 vol./vol.% for the essential oil. In both cases, IZD increased with concentration. However, the extract consistently produced larger inhibition zones, demonstrating greater antifungal potency. The maximum IZD reached 26.63 mm at 40 wt.% extract, nearly double that of the essential oil (13.69 mm at 40 vol./vol.%), confirming that the extract form of *P. betle* exhibited markedly stronger antifungal activity than its essential oil counterpart. Kumpanich et al. [56] confirmed antifungal activity of *P. betle* extract. However, they reported smaller IZD values (7.58, 10.43, and 16.40 mm for 5, 10, and 20 wt./wt.%, respectively, which may be attributed to variations in extract composition, incubation time, or culture medium used.

Rajali et al. [49] found dose-dependent antifungal activity of *O. basilicum* oil in Visco-gel (A-SLM, TC, Table 1 and Table 4). At 0.25 mL, corresponding to the minimal fungicidal concentration (MFC), biofilm formation was 55.06% on day 1 and 52.68% on day 14, compared with 60.03% and 65.94% at the lower 0.0625 mL concentration, corresponding to the MIC. These results revealed the sustained antifungal action of *O. basilicum* oil over time and distinguished between inhibitory and fungicidal effects. Such differentiation is valuable, as the choice of concentration depends on whether the goal is to inhibit fungal proliferation or achieve complete fungal eradication.

Abdallah et al. [55] measured concentration-dependent IZDs (6 and 8.2 mm) for *C. longa* (10 and 20 vol./vol.% ethanolic curcumin extracts, respectively) in Trusoft (A-SLM, TC, Table 1 and Table 4).

Ahmed et al. [54] reported IZDs of 12.56 and 16.72 mm for Vertex Soft (A-SLM, ST-SLM, Table 1 and Table 4) loaded with *Cinnamomum* oil (1 and 2 wt.%), confirming a clear concentration-dependent antifungal effect.

Memon et al. [40] demonstrated 80% reduction in fungal cell count for 2 wt.% *A. vera* powder in GC Soft Liner (A-SLM, ST-SLM, Table 1 and Table 4), indicating strong fungicidal potential even at low concentrations.

Finally, chitosan is included in this review as a specific antifungal additive for SLMs, distinct from plant oils and extracts. Unlike phytochemicals, whose activity depends largely on bioactive molecules, chitosan exerts its antifungal effect through its polycationic structure. It causes fungal membrane disruption due to the electrostatic interactions between positively charged quaternary ammonium groups of chitosan with the negatively charged fungal cell membrane [38,39].

Mohammed et al. [57] demonstrated that Vertex Soft (A-SLM, ST-SLM, Table 1 and Table 4) loaded with chitosan nanoparticles (1.5 and 2 wt.%) reduced fungal adhesion by more than 50 and 70%, respectively. Memon et al. [40] obtained similar result for GC Soft Liner (A-SLM, TC, Table 1 and Table 4) modified with 2 wt.% chitosan, observing a 50% reduction in *C. albicans* cells. Saeed et al. [58] further compared two forms of chitosan incorporated into GC Soft Liner: commercial (MIC = 0.625 mg/mL) and synthesized (MIC = 0.3125 mg/mL). Samples containing commercial chitosan significantly inhibited *C. albicans* growth for 24 h immersion in artificial saliva. In contrast, SLMs modified with lower-MW chitosan maintained significant inhibition up to 3 days. The stronger activity of synthesized chitosan was attributed to its lower molecular weight, which likely improved dispersion within the SLM matrix. No substantial difference was observed between the 2 times and 4 times MIC values, indicating that the lower concentration was sufficient to achieve effective antifungal activity.

Overall, the antifungal activity of modified SLMs showed clear dose-, time-, and plant-dependent relationships. Both immersion and direct incorporation methods proved effective, with silicone-based SLMs generally enhancing additive release. Specific combinations of plasticizer and solvent further enhance efficacy, suggesting promising directions for multifunctional bioactive SLMs. These findings also support the development of advanced delivery strategies, including microparticle incorporation, microencapsulation, and nanoformulations, to protect bioactive compounds from premature degradation, provide sustained release, improve solubility and diffusion of hydrophobic substances, and achieve more uniform distribution, ultimately enhancing antifungal performance against *C. albicans* [36]. Further studies are needed to explore higher concentrations or alternative formulations to maximize the antifungal potential of SLMs.

However, wide heterogeneity in experimental protocols and evaluation criteria of antifungal testing produce a bias. Generally, bias arises from variations in SLM type, composition, additive incorporation method, and concentration, all of which affect additive distribution and release behaviour. Although *C. albicans* was consistently used across studies, major differences in testing methodology and material preparation limit comparability. Testing media (e.g., Sabouraud dextrose agar vs. tryptic soy broth) influenced fungal growth and additive diffusion, while inconsistent definitions of minimum inhibitory and fungicidal concentrations further reduced cross-study consistency. The most frequently investigated bioactives were *M. alternifolia*, *A. indica*, and chitosan, whereas other plant species were tested only once or twice, hindering comparative interpretation. Exposure durations were typically unstandardized, influencing sustained antifungal activity. Most studies used water rather than saliva, failing to replicate intraoral conditions such as pH variation, temperature changes, or mechanical stress. Finally, the common reliance on IZD without quantitative validation (e.g., CFU counts or biofilm assays) also introduced significant measurement bias.

### 5.2. In Vivo Antifungal Efficacy

Ojah et al. [77] conducted the only in vivo study evaluating the antifungal effects of *A. indica*, Triphala, and *A. vera* on relined dentures (SLM type not specified) to assess antifungal effect against *C. albicans*. 10 participants of the study either immersed their dentures overnight in aqueous solutions of *A. indica* or Triphala, or rubbed them with *A. vera* leaves every other day for 15 days. Microbiological analysis showed that *A. indica* immersion produced the most pronounced effect, achieving a 71% reduction in *C. albicans* counts compared to pre-treatment levels, which corresponded to the efficacy comparable to commercial denture-cleaning tablets. Triphala immersion yielded a 51% reduction, while *A. vera* treatment resulted in only a 41% decrease. These findings indicate that *A. indica* provided the strongest in vivo antifungal efficacy among the tested plant-derived additives, whereas *A. vera* exhibited only modest antifungal potential under the same conditions (Table 5).

In vivo evidence for SLMs modified with plant-derived additives is very limited, with only one study available. This study had a small sample size and lacked randomization or blinding. The SLM was not specified, and application methods varied, such as immersion versus topical use, leading to inconsistent exposure. Microbial assessments relied on colony counts and did not account for specific oral factors. These limitations make it difficult to generalize the results and highlight the need for well-designed, controlled clinical trials.

### 5.3. In Vitro Antibacterial Efficacy

The antibacterial potential of commercially available SLMs modified with plant-derived additives, in the form of essential oils, solutions, or liquid and dry extracts, has also been investigated. In total, 5 plant-derived species were identified. The most frequently tested were *A. indica* and *M. alternifolia*. The remaining, each evaluated once, included *P. betle*, *L. cubeba*, and *O. vulgare*. Antibacterial activity was primarily evaluated against *Streptococcus mutans* (5 studies). Other tested bacteria strains included: *Staphylococcus aureus* (3 studies), *Escherichia coli* (3 studies), *Pseudomonas aeruginosa* (1), *Streptococcus sanguis* (1), and *Bacillus subtilis* (1) (Table 6).

Singhania et al. [61] incorporated *A. indica* extract into GC Soft Liner (A-SLM, TC, Table 1 and Table 6) and reported strong antibacterial activity against *S. mutans* (log CFU/mL = 2.14) and *S. aureus* (log CFU/mL = 2.44), comparable to chlorhexidine-modified material (log CFU/mL = 2.04 for *S. mutans* and 3.10 for *S. aureus*). However, *A. indica* exhibited limited efficacy against *E. coli* (log CFU/mL = 6.22), indicating reduced activity toward Gram-negative bacteria. Given its dual antifungal and antibacterial effects, *A. indica* appears to be a promising additive for preventing oral candidiasis.

Poolkerd et al. [34] compared *P. betle* extract and essential oil incorporated into GC Soft Liner (A-SLM, TC, Table 1 and Table 6) at concentrations ranging from 2.5 to 70 wt./wt.% and 2.5 to 70 vol./vol.%, respectively. Both formulations exhibited dose-dependent inhibition of *S. mutans*. Growth inhibition zones were first observed at 10 wt.% for the extract (IZD = 8.38 mm) and at 60 vol./vol.% for the essential oil (IZD = 8.31 mm). The IZD increased with concentration, reaching 26.76 mm at 70 wt./wt.% extract and 9.30 mm at 70 vol./vol.% essential oil. These findings demonstrate that the *P. betle* extract exhibited markedly higher antibacterial activity than its essential oil counterpart. However, both formulations showed weaker antibacterial than antifungal potency.

AlHamdan et al. [62] reported significant antibacterial of GC Soft Liner (A-SLM, TC, Table 1 and Table 6) immersed in *M. alternifolia* oil for 48 h against *E. coli*, *S. aureus*, and *S. mutans* (log CFU/mL of 2.45, 2.21, and 2.33, respectively). These results were comparable to those of chlorhexidine-modified GC Soft Liner (2.14, 3.10, and 2.04, respectively), indicating that *M. alternifolia* oil exerts a broad-spectrum inhibitory effect against both Gram-positive and Gram-negative bacteria, supporting its potential as a natural alternative to chlorhexidine [62].

Songsang et al. [53] studied *L. cubeba* oil (5–30 vol./vol.%) in three acrylic-based TCs: Visco-gel, GC Soft Liner, and Coe-Comfort (A-SLM, Table 1 and Table 6). Antibacterial activity against *S. mutans* appeared only at 30 vol./vol.%, with similar IZDs (7.89 mm for GC Soft Liner, 7.96 mm for Visco-gel, and 8.15 mm for Coe-Comfort), indicating neglecting influence of SLM formulation on antibacterial performance. These values were approximately 60% smaller than those of the 2% chlorhexidine control, indicating limited potency.

Baygar et al. [59] evaluated carvacrol (10 mL), the main component of *O. vulgare* oil, incorporated into silicone-based Ufi Gel P (S-SLM, LT-SLM, Table 2 and Table 6). Strong antibacterial effects were observed, decreasing in the order: *B. subtilis* (IZD = 43.67 mm) > *S. mutans* (IZD = 40.33 mm) > *S. sanguis* (IZD = 36.67 mm) > *S. aureus* (IZD = 34.00 mm) for Gram-positive bacteria, and *E. coli* (IZD = 29.33 mm) > *P. aeruginosa* (IZD = 15.33 mm) for Gram-negative. MTT assay results supported these findings, indicating 90–99% biofilm inhibition in Gram-positive strains, moderate inhibition in *E. coli* (70%), and lower sensitivity in *P. aeruginosa* (68%). These results confirm the broad-spectrum antibacterial potential of carvacrol, with particularly strong activity against Gram-positive bacteria.

In summary, *A. indica*, *M. alternifolia*, *L. cubeba*, *P. betle*, and carvacrol, demonstrate both antifungal and antibacterial activity, indicating dual protection of SLMs against oral pathogens. Gram-positive bacteria are generally more susceptible than Gram-negative species, and silicone-based SLMs may show higher activity due to hydrophobic character enhancing diffusion of lipophilic additives.

Bias in antibacterial testing arises from substantial variability in study design, with most research conducted on GC Soft Liner and only a few studies evaluating other SLMs. Differences in plant species, additive concentrations, incorporation methods, and target microorganisms make direct comparisons difficult. Testing often focuses on a limited set of bacterial species, and results vary with bacterial strain and SLM composition. The lack of standardized protocols and limited long-term evaluations further reduces the reliability and generalizability of reported antibacterial effects.

### 5.4. Biocompatibility

Biocompatibility testing of SLMs modified with plant-derived additives has been limited to 2 in vitro studies on *L. cubeba* and *P. betle*. Songsang et al. [53] evaluated Visco-gel, GC Soft Liner, and Coe-Comfort (A-SLMs, TCs, Table 1) incorporated with 10 and 30 vol./vol.% *L. cubeba* essential oil using the MTT assay on human gingival fibroblast cells for 24 h. Cell viability remained above 70%, indicating non-toxicity. Similarly, Poolkerd et al. [34] tested GC Soft Liner loaded with 5 and 10 wt./wt.% *P. betle* extract, finding cell viability greater than 90%, also confirming non-cytotoxicity.

To complement the limited biocompatibility data, LD_50_ values of selected natural compounds (Table 7) were compared with those of conventional antifungal drugs and antiseptics (Table 8).

As shown in Table 7, most natural remedies have high LD_50_ values (>5000 mg/kg), classifying them as “practically non-toxic” according to WHO criteria [137]. Exceptions include *O. sanctum* oil (4.57 mg/kg) and *A. sativum* ethanol extract (4.47 mg/kg), which are considered “highly hazardous” at low doses. Toxicity depends on the form of a plant-derived additive, with essential oils generally more toxic than aqueous or methanolic extracts, test species, and whether whole extracts or isolated compounds are used (e.g., *N. sativa* oil vs. thymoquinone).

Compared to conventional antifungal drugs and antiseptics (Table 8), natural remedies generally appeared safer. Common agents such as ketoconazole (166 mg/kg in rats) are highly toxic, while fluconazole, clotrimazole, and chlorhexidine are moderately hazardous. Nystatin exhibits species-dependent toxicity: highly in mice (200 mg/kg) but low in rats (10,000 mg/kg). These comparisons highlight that plant-derived additives appear to offer antifungal effects with lower acute toxicity, suggesting a safer alternative to synthetic drugs.

The low cytotoxicity reported for *L. cubeba* (Songsang et al. [53]) and *P. betle* (Poolkerd et al. [34]) in vitro align with in vivo LD_50_ data (*L. cubeba*: 4000 mg/kg in mice [116], >5000 mg/kg in rats [117], and *P. betle* methanolic extract: >5000 mg/kg in mice [125]), supporting their general safety for SLM formulations. The findings suggest that any observed cytotoxicity in modified SLMs is more likely due to the unreacted methacrylate monomers, plasticizers, or polymerization initiators released from the material rather than to the plant-derived additives themselves [138,139,140].

Bias in biocompatibility assessment arises from several methodological limitations. Few studies have evaluated cytotoxicity, typically using short-term assays on a single cell type, which restricts clinical relevance despite generally acceptable cell viability results. Variations in additive concentration, extraction solvents, and SLM formulations further reduce comparability among studies. The absence of complementary assays and long-term or multi-cell evaluations limits understanding of biological safety, while the lack of standardized testing protocols contributes to inconsistent outcomes. To ensure reliability, broader and standardized biocompatibility assessments, including incorporating multiple cell types, extended exposure periods, and simulated oral conditions, are necessary to confirm the biological safety of plant-modified SLMs.

Overall, there is a substantial gap in biocompatibility data for plant-modified SLMs, highlighting the need for comprehensive in vitro and in vivo testing.

### 5.5. Intrinsic Mechanical Properties

The next issue concerns how incorporating plant-derived additives affects the intrinsic mechanical properties of SLMs. Additives may alter hardness, tensile strength, and tear resistance, potentially compromising durability or structural integrity. Therefore, systematic testing is needed to ensure that antifungal or antibacterial enhancements do not negatively impact the SLM’s ability to withstand chewing, insertion, and removal forces, maintaining both clinical performance and antimicrobial efficacy.

The influence of 11 plant-derived additives: *M. alternifolia* (1 study), *C. citratus* (1 study), *T. vulgaris* (2 studies), *A. sativum* (1 study), *A. indica* (2 studies), *P. betle* (1 study), *L. cubeba* (1 study), *C. nucifera* (1 study), *Cinnamomum* (1 study), *S. indicum* (1 study), and *A. vera* (2 studies), on the mechanical properties of SLMs was examined. The following properties were assessed: Shore A hardness (6 studies), tensile strength (1 study), and tear strength (2 studies) (Table 9).

Hardness is a key mechanical property of SLMs. It determines the balance between cushioning comfort and dimensional stability. Optimal hardness ensures that the SLM is soft enough to absorb masticatory stresses and adapt to underlying tissues, yet firm enough to resist excessive deformation and wear. Variations in hardness may arise from plasticizer leaching, water absorption, or chemical modification of the SLMs. Incorporation of plant-derived additives can further alter hardness, either increasing it by reducing plasticization or decreasing it through enhanced flexibility or water uptake. Maintaining appropriate hardness is therefore essential to preserve both the functional and clinical performance [41,53].

The ISO:10139-1:2018 standard specifies the Shore A hardness of soft and very soft SLMs after 2-h and 7-day storage of samples in distilled water. SLM is defined as “soft” when its Shore A hardness is in the range from 30 to 50 °Sh and higher than 60 °Sh after 2 h and 7 days, respectively, and “extra soft” when these values are lower than 30 °Sh and 60 °Sh, respectively [141]. For clinical use of SLMs, a Shore A hardness is required to be from 13 to 49 °Sh at 24 h [142].

Several studies have evaluated the effect of plant-derived additives on the Shore A hardness of GC Soft Liner (TC, Table 1 and Table 9). The results revealed that both the type and concentration of the plant-derived additive, as well as immersion time influence this property.

Patil et al. [63] reported that incorporation of 50 vol./vol.% *C. citratus*, *M. alternifolia*, and *T. vulgaris* essential oils in GC Soft Liner (Table 1 and Table 9) did not significantly alter hardness after 24 h in artificial saliva, indicating good compatibility between the oils, the butyl phthalate butyl glycolate plasticizer, and PMMA. After 60 days, hardness increased for both unmodified and modified samples, consistent with ethanol and plasticizer leaching [143,144,145]. The neat GC Soft Liner (51 °Sh) showed a 16% rise in hardness, while the hardness of modified samples increased by 5%, 14%, and 18% for *M. alternifolia*, *C. citratus*, and *T. vulgaris*, respectively. This suggests decreasing compatibility in the order: *M. alternifolia* > *C. citratus* > *T. vulgaris*. The relatively small hardness changes observed for *M. alternifolia* oil suggest effective plasticization and, consequently, good long-term stability. The minimal change for *M. alternifolia* indicates effective plasticization and long-term stability, though possibly reduced antibacterial activity due to limited leaching of bioactive components.

Kumar et al. [44] found that incorporation of *A. sativum* and *A. indica* significantly increased hardness. After 2 days, *A. sativum* and *A. indica* caused 30% and 41% increases relative to the neat GC Soft Liner (10 °Sh), while over 7 days, hardness increased by 15% and 40%, respectively. The initial increases can be attributed to limited compatibility between the bioactive compounds with the plasticizer and PMMA, while subsequent stiffening reflects the leaching of bioactive compounds [144,145]. The stronger effect observed for *A. indica* is likely due to the certain water solubility of azadirachtin, nimbidin, and gedunin, which promotes faster leaching. In contrast, *A. sativum*, containing the less polar and practically water-insoluble allicin, exhibited greater compositional stability over time.

Conversely, Kumpanich et al. [56] demonstrated a clear concentration-dependent softening effect of *P. betle* extract on hardness. Incorporation of 5, 10, and 20 wt./wt.% extract led to hardness reductions of 5%, 35%, and 65% after 2 h, and 12% and 42% after 7 days for medium and high concentrations, respectively. Interestingly, a slight hardness increase (4%) occurred at 5 wt.% after 7 days, suggesting mild re-stiffening at low additive levels. Thus, *P. betle* acts as a stiffener at low concentrations but as a plasticizer at higher ones. The trend, in which hardness decreases at low plasticizer concentrations but stabilizes or even increases at higher concentrations, can be explained by specific polymer–plasticizer interactions. At low levels, plasticizers solvate polymer chains, weakening intermolecular forces and increasing free volume, thereby softening the material. At higher concentrations, incompatibility may occur, leading to phase separation and reduced plasticizing efficiency [146].

Overall, these studies collectively demonstrate that plant-derived additives can modulate the hardness of GC Soft Liner in distinct ways, softening when acting as compatible plasticizers (e.g., *P. betle*, *L. cubeba*, *C. citratus*) and hardening when inducing phase incompatibility or promoting plasticizer leaching (*A. indica*, *A. sativum*). Thus, the chemical nature and polarity of bioactive compounds, particularly their water solubility and affinity for the plasticizer/polymer system, are key determinants of long-term hardness stability in bioactive SLMs.

Songsang et al. [53] compared the effect of *L. cubeba* oil (5–30 vol.%) on the Shore A hardness of three TCs (A-SLMs, Table 1 and Table 9): GC Soft Liner, Coe-Comfort, and Visco-gel after 2 h and 7 days of water immersion. In all cases, incorporation of *L. cubeba* oil reduced hardness, with greater scale at higher concentrations. After 2 h, the hardness reduction was most pronounced for Coe-Comfort (14–87%, with 16.32 °Sh for the neat TC), followed by GC Soft Liner (32–81%, with 21.72 °Sh for the neat TC), and least for Visco-gel (from 7 to 40%, with 37.13 °Sh for the neat TC). These differences can be explained by the varying compatibility between citral (main bioactive component in *L. cubeba* oil) and the plasticizers. Strong citral–plasticizer interactions in GC Soft Liner (butyl phthalate butyl glycolate) and Coe-Comfort (benzyl benzoate), likely increased polymer chain mobility, producing greater reduction in hardness. In contrast, Visco-gel (triethyl citrate) showed weaker softening, indicating lower citral-plasticizer compatibility [144]. After 7 days, overall hardness increased across all samples due to leaching phenomena [144,145]. However, the concentration-dependent softening trend persisted (10–77% for Coe-Comfort, 13–65% for GC Soft Liner, and 6–31% for Visco-gel). These results confirm that both SLM composition and additive concentration strongly affect mechanical response of SLM.

For Vertex Soft (ST, A-SLM, Table 1 and Table 9), two studies provided complementary insights. Bushra et al. [52] demonstrated that incorporating *C. nucifera* oil reduced Shore A hardness by up to 28%, confirming its effective plasticization. The softening effect was stronger at 1.5 vol.% than at 2.5 vol.%, consistent with previous findings [56] that higher additive levels can cause incompatibility and phase separation [146]. *C. nucifera* oil, rich in medium-chain fatty acids [21,22], likely acts as an efficient plasticizer at lower concentrations but less so at higher ones, due to reduced miscibility with acetyl tributyl citrate. Similarly, Ahmed et al. [54] observed a slight hardness increase (4–7%) upon addition of *Cinnamomum* oil (1–2 wt.%), attributed to limited miscibility of its polyphenolic components (e.g., cinnamaldehyde, curcumin, gingerol) with the same plasticizer.

Hatim et al. [48], Abdulwahhab et al. [41] and Noori et al. [42] extended mechanical evaluations of Vertex Soft (ST, A-SLM, Table 1 and Table 9) by assessing tensile, deformation, and tear resistance, which are key indicators of cohesive integrity and durability under clinical stresses [147,148]. Hatim et al. [48] tested tensile strength and percentage elongation for *S. indicum* and *T. vulgaris* oils (5 vol.% individually and 2.5 vol.% each in combination) after the SLM immersion in water for up to 30 days. The neat Vertex Soft exhibited stable tensile strength (4.5–4.9 MPa) and elongation (5%) over time. All modified formulations showed improved strength, most notably for *T. vulgaris* (18% after 2 days and 25% after 30 days), followed by the mixture (15 and 20%) and *S. indicum* (10 and 14%). Elongation increased similarly, indicating enhanced flexibility. These improvements result from the plasticizing action of the oils, which increase polymer chain mobility, while later deterioration reflects leaching and water absorption [144,145,146].

Abdulwahhab et al. [41] and Noori et al. [42] investigated tear strength in Vertex Soft (Table 1 and Table 9). Incorporating 3 wt.% *A. vera* increased tear strength by approximately 5% after both 24 h and 4 weeks of storage in artificial saliva, whereas 10 wt.% reduced it by about 5% and 6%. Similarly, Noori et al. [42] reported that adding 10 wt.% *A. indica* and *A. vera* increased tear strength by approximately 2% and 16%, respectively. These results indicate that moderate concentration of plant-derived additives can enhance tear resistance without compromising the mechanical integrity of the SLM. The observed increase in tear strength over time can be attributed to the leaching of less compatible molecules, which strengthens polymer cohesion.

Bias in evaluating the intrinsic mechanical properties of SLMs arises from several methodological limitations. The number of studies is small, most focusing on Vertex Soft and GC Soft Liner, with few data on other materials and no studies on S-SLMs. Only a limited set of properties has been evaluated, most frequently hardness, which is important, while tensile strength, elongation, and tear strength have typically been assessed in only one study each. Differences in SLM type, additive form, concentration, and modification method can create local heterogeneity and air entrapment, affecting results. Variations in the gelation process, sample geometry, and experimental parameters further influence measurements. Small sample sizes limit reproducibility and generalizability. Finally, lack of standardized protocols makes it difficult to distinguish true material behavior from artifacts introduced during preparation or testing.

### 5.6. Interface Mechanical Properties

The interface mechanical properties play a crucial role in determining the clinical performance and longevity of denture bases lined with SLMs. High peel strength prevents delamination and micro-gap formation during insertion, removal, and mastication. Adequate shear strength resists lateral forces that could shift or tear the liner, and sufficient tensile bond strength maintains attachment under forces perpendicular to the interface [149,150].

The influence of five plant-derived additives on SLMs interface mechanical properties has been investigated: *A. vera* (2 studies), *C. citratus* (1 study), *A. indica* (1 study), and *Cinnamomum* (1 study). The interface mechanical properties tested included shear bond strength (2 studies), peel bond strength (1 study), and tensile bond strength (1 study) (Table 10).

Naser et al. [51] evaluated the effect of *C. citratus* oil on peel bond strength by incorporating 2.5 and 5 vol.% into Moonstar Soft (ST-SLM, Table 1 and Table 10). Peel bond strength decreased by 15% and 23% for 2.5 and 5 vol.% *C. citratus* oil, respectively, compared with the unmodified material (2.39 MPa). The reduction in peel bond strength can be attributed to phase separation caused by weak compatibility between citral—the main component of *C. citratus* oil, plasticizer, and polymer matrix (dibutyl phthalate and PEMA, respectively). This incompatibility likely limited proper solvation of the polymer chains, resulting in the formation of separate domains that created weak points in the interface, facilitating detachment of the SLM from the acrylic denture base.

For Vertex Soft (TC, Table 1 and Table 10) shear bond strength increased with the incorporation of plant-derived additives. Noori et al. [43] reported that 10 wt.% *A. indica* increased shear bond strength by 26.1%, while 10 wt.% *A. vera* produced an 8.1% increase relative to the neat Vertex Soft (0.444 MPa). The improvement was attributed to the smaller particle size of *A. indica* (2–5 µm) and *A. vera* (3–6 µm) compared with Vertex Soft powder (15–46 µm), as well as efficient solvation arising from strong compatibility between the bioactive components (azadirachtin, nimbidin, gedunin in *A. indica*, and acemannan, aloemannan in *A. vera*) and the polymer–plasticizer system (PEMA-acetyl tributyl citrate). Similarly, Abdulwahhab et al. [41] observed that 3 wt.% *A. vera* increased shear bond strength by 45% after 24 h and 200% after 4 weeks, whereas 10 wt.% produced slightly smaller increases (40% at 24 h, 180% at 4 weeks).

These findings indicate that moderate loading of plant-derived additives can enhance polymer–plasticizer solvation, promote gelation, and interfacial adhesion, with *A. indica* providing stronger effect.

Abdallah et al. [55] investigated the effect of *C. longa* extract added to Trusoft (TC, Table 1 and Table 10) at concentrations of 10 and 20 vol./vol.% on tensile bond strength. The unmodified SLM showed a tensile bond strength of 0.4 MPa, whereas incorporation of 10 and 20 vol./vol.% *C. longa* extract increased the strength by 23% and 27%, respectively. This improvement can be attributed to the plasticizing effect of phenolic compounds and flavonoids in *C. longa*, which enhanced surface adaptation to the prosthesis and potentially increased the interaction between the *C. longa*-modified SLM and the denture base.

In summary, the addition of bioactive plant-derived components to SLMs can have contrasting effects on interface properties, depending on their compatibility with the polymer–plasticizer system. *C. citratus* oil reduced peel bond strength, whereas moderate incorporation of *A. indica*, *A. vera*, and *C. longa* generally enhanced shear and tensile bond strength. Optimal additive concentrations are critical for maximizing interface strength while avoiding phase incompatibility. Overall, these findings emphasize the importance of balancing bioactivity and mechanical compatibility to maintain SLM performance at the denture–liner interface. However, the high diversity of SLMs and plant species (each study used a different plant-derived additive as well as SLM) potentially introduces substantial bias and limits the ability to generalize findings across SLM types or bioactive agents. Differences in additive concentration, modification technique, and gelation conditions further contribute to variability in results.

### 5.7. Roughness

Surface roughness is a critical property of SLMs, as it directly affects microbial adhesion, plaque accumulation, and oral tissue health. Smoother surfaces are preferred, as increased roughness promotes adhesion and proliferation of pathogenic microorganisms. Roughness is affected by SLM composition, exposure to cleaning agents, and the homogenization process, during which air bubble entrapment and migration can create surface cavities [151,152].

The following plants were each tested once for their influence on the surface roughness of SLMs: *C. anthelminticum*, *O. sanctum*, *L. usitatissimum*, *C. citratus*, *Cinnamomum*, and *O. basilicum*.

Visco-gel (A-SLM, TC, Table 1 and Table 11) was investigated twice for surface roughness modification with plant-derived additives. Muttagi et al. [46] found that incorporation of 800 µL of *C. anthelminticum* and *O. sanctum* oils produced smooth surface layers, with *O. sanctum* being more uniformly distributed. However, smoother surfaces did not correlate to stronger antifungal effects, as *C. anthelminticum* showed superior activity. In contrast, Rajali et al. [49] found that adding 5 vol.% *O. basilicum* oil slightly increased surface roughness, suggesting that higher additive concentrations may disturb matrix uniformity.

Similarly, Naser et al. [51] reported that *C. citratus* oil (2.5 and 5 vol.%) reduced surface roughness of Moonstar Soft (TC, A-SLM, Table 1 and Table 11) by up to 30%, attributed to improved polymer chain organization during gelation. In line with this, Abdallah et al. [55] found that adding higher concentrations (10 and 20 vol./vol.%) of *C. longa* extract into Trusoft (TC, Table 1 and Table 11) decreased surface roughness by up to 36%, confirming that plant-derived additives can improve surface uniformity when present in optimum amounts.

Overall, studies show that plant-derived additives generally reduce surface roughness of SLMs. The effect, however, depends strongly on the plant species, concentration, and compatibility with the polymer matrix. Moderate concentrations, such as with *C. citratus* or *C. longa*, improve surface smoothness, whereas excessive amounts, as observed with *O. basilicum*, may disrupt matrix uniformity. Optimizing additive type and concentration is therefore essential to balance surface properties, antifungal performance, and material integrity.

Bias in surface roughness assessment arises from the small number of studies and the frequent use of qualitative instead of quantitative methods. Variations in additive concentration, modification method, and gelation process further limit comparability, reducing confidence in the reported outcomes.

### 5.8. Prospects and Future Directions

SLMs modified with plant-derived additives present significant industrial potential due to their antimicrobial properties and favorable physico-mechanical performance. Increasing global interest in medicinal plants and phytochemicals has promoted their use as natural components in prosthetic dental materials. The international trade of medicinal plants, including essential oils, tannins, and botanical extracts, has experienced remarkable growth, rising from USD 33 billion in 2014 [153] to USD 410.3 billion in 2024 and projected to reach USD 890.7 billion by 2034 [154]. According to Vasisht et al. [153], the global market for medicinal plants is expected to outpace that of conventional drugs.

The advantages of plant-derived compounds have been highlighted by Camaioni et al., including their high purity and standardization (≥95%), low cost-effectiveness, with prices of less than 1 EUR/g, and the presence of reactive functional groups (such as hydroxyl, carboxylic acid, or ketone), enabling their use in drug delivery systems. However, these advantages generally apply to moderately processed or purified complex heterogeneous compositions rather than to specific bioactive substances.

While these trends highlight substantial economic opportunities, the industrial-scale production of SLMs incorporating plant-derived fungicides faces significant feasibility challenges, particularly due to the high cost and technical complexity of purifying and isolating individual bioactive compounds, as well as issues related to standardization and sustainable sourcing of plant materials.

Zamani et al. [155] note that variability in plant quality, lack of standardization, and limited scientific validation can lead to inconsistent antifungal efficacy and complicate material formulation. Additionally, Camaioni et al. [156] highlight issues such as the complexity of plant mixtures, low natural abundance of some compounds, uncertainties regarding bioavailability and toxicity, and ethical or environmental considerations related to sourcing from endangered species.

An additional challenge observed in this review is the trade-off between antimicrobial efficacy and polymer stability during prolonged clinical use. High concentrations of plant-derived additives can enhance antifungal or antibacterial activity but may also accelerate plasticizer leaching, alter hardness, or compromise mechanical integrity over time. Future research should focus on optimizing the type and concentration of additives to achieve a balance between sustained antimicrobial performance and long-term durability of SLMs.

Looking ahead, the future perspectives for SLMs with plant-derived additives are promising. Camaioni et al. [156] noted that these compounds would be utilized in next-generation antifungal drugs. Particularly, pure bioactive compounds offer a standardized and scalable alternative to complex extracts, improving reproducibility and sustainability in SLM modification. Zamani et al. [155] further emphasize that addressing critical issues, such as global standardization, sustainable sourcing, and quality assurance, will strengthen the medicinal plant sector. The rising global demand for natural and organic compounds, combined with technological advances in dentistry, is expected to drive the development of high-quality, productive antimicrobial SLMs enhanced with plant-derived additives.

## 6. Conclusions

Plant-derived additives have emerged as promising alternatives for modifying SLMs due to their potential antifungal activity and generally favorable safety profile. These compounds have been incorporated into SLMs by addition to either the powder or liquid component, direct mixing into the uncured formulation, or through post-curing immersion. Their incorporation often enhanced mechanical properties and roughness depending on compatibility with the polymer–plasticizer system.

Across the reviewed studies, several plant-derived species were identified, including: *M. alternifolia*, *A. indica*, *A. vera*, *A. sativum*, *O. vulgare*, *T. vulgaris*, *O. basilicum*, *O. sanctum*, *C. nucifera*, *Cinnamomum*, *C. longa*, *Z. officinale*, *N. sativa*, *L. cubeba*, *C. citratus*, *L. usitatissimum*, *C. anthelminticum*, *P. betle*, and chitosan. Among these, only *A. indica*, *M. alternifolia*, and chitosan demonstrated consistent in vitro antifungal effects against *C. albicans* across at least three independent studies. Antimicrobial activity generally exhibited dose- and time-dependent behavior, with *A. indica* showing stronger fungicidal activity than *M. alternifolia*.

However, the available evidence is constrained by methodological variability and potential biases. Variations in additive concentration, extraction method, additive form (liquid, extract, solution, or powder), and mode of incorporation (direct addition or immersion) contribute to inconsistent findings. Most plant-derived additives were examined in only a few studies, often focusing solely on antimicrobial activity without accompanying assessments of biocompatibility or mechanical performance, resulting in fragmented, difficult-to-compare data. Many investigations also relied on qualitative assessments without quantitative validation. Additionally, natural variability in plant extract composition, affected by extraction solvents, geographical origin, and seasonal factors, further compromises reproducibility. Collectively, these limitations underscore the need for standardized methodologies, comprehensive reporting, and rigorous validation in future research.

Nevertheless, the high therapeutic potential and growing commercial relevance of these natural compounds justify further systematic research, emphasizing the need for standardized, sustainable, and clinically oriented development.

## Figures and Tables

**Table 1:** Comparative overview of acrylic-based SLMs (TCs and ST-SLMs) modified via addition method.

Plant Latin Name (Common Name) (Family)	Additive Content (Form)	SLM Type/Name (Manufacturer, Country) Powder/Liquid Ratio	Antimicrobial Properties	Reference
	Enriched SLM Component			
			Mechanical Properties	
*Aloe vera* (aloe) (Liliaceae)	2 wt. % (powder)	TC/GC Soft Liner (GC Corp., Tokyo, Japan) 1.2 g/1 mL	*C. albicans* colony count	[40]
	powder		-	
	3 and 10 wt.% (powder)	ST-A-SLM/Vertex Soft (Vertex Dental, Soesterberg, The Netherlands) 1.2 g/1 mL	-	[41]
	powder		Tear strength, shear bond strength	
	10 wt.% (powder)	ST-A-SLM/Vertex Soft (Vertex Dental, Soesterberg, The Netherlands) 1.2 g/1 mL	-	[42,43]
	formulation		Tear strength, shear bond strength	
*Azachirachta* *indica* (Neem) (Meliaceae)	50, 100, 200, 400, 500 µg (powder)	TC/GC Soft Liner (GC Corp., Tokyo, Japan) 1.2 g/1 mL	*C. albicans* colony count	[44]
	powder		Shore A hardness	
	5, 10, 15, 20, 25, 30, 35, 40 vol./vol. % (oil extract)	TC/Visco-gel (Dentsply DeTrey, Konstanz, Germany) 3 g/2.2 mL	*C. albicans* inhibition zone	[45]
	liquid		-	
	10 wt.% (powder)	ST-A-SLM/Vertex Soft (Vertex Dental, Soesterberg, The Netherlands) 1.2 g/1 mL	-	[42,43]
	formulation		Tear strength, shear bond strength	
*Linum usitatissimum* (flaxseed) (Linaceae)	800, 900 and 1000 µL (seed oil)	TC/Visco-gel (Dentsply DeTrey, Konstanz, Germany) 3 g/2.2 mL	*C. albicans* inhibition zone	[46]
	liquid		Roughness	
*Centratherum* *anthelminticum* (bitter cumin) (Asteraceae)	600, 700 and 800 µL (seed oil)	TC/Visco-gel (Dentsply DeTrey, Konstanz, Germany) 3 g/2.2 mL	*C. albicans* inhibition zone	[46]
	liquid		Roughness	
*Origanum vulgare* (oregano) (Lamiaceae)	50 µg/mL (oil)	TC/Acrosoft (Marlik Medical Industries Co., Tehran, Iran) 3.2 g/2.5 mL	*C. albicans* inhibition zone	[47]
	formulation		-	
*Thymus vulgaris* (thyme) (Lamiaceae)	5 vol.% (oil)	ST-A-SLM/Vertex Soft (Vertex Dental, Soesterberg, The Netherlands) 1.2 g/1 mL	-	[48]
	liquid		Tensile strength	
*Ocimum sanctum* (Holy Basil and Tulsi) (Lamiaceae)	600, 700 and 800 µL (oil)	TC/Visco-gel (Dentsply DeTrey, Konstanz, Germany) 3 g/2.2 mL	*C. albicans* inhibition zone	[46]
	liquid		Roughness	
*Ocimum basilicum* (basil) (Lamiaceae)	1.25 and 5 vol./vol. % (oil)	TC/Visco-gel (Dentsply DeTrey, Konstanz, Germany) 3 g/2 mL	*C. albicans* biofilm formation	[49]
	liquid		Roughness	
	5, 10, 15, 20, 25, 30, 35, 40 vol./vol. % (oil)	TC/Visco-gel (Dentsply DeTrey, Konstanz, Germany) 3 g/2 mL	*C. albicans* inhibition zone	[45]
	liquid		-	
*Melaleuca* *alternifolia* (tea tree) (Myrtaceae)	0.5, 1, 1.5, 2 mL (10, 20, 30, 40 vol./vol. % oil extract)	TC/Visco-gel (Dentsply DeTrey, Konstanz, Germany) 3 g/2.2 mL TC/GC Soft Liner (GC Corp., Tokyo, Japan) 1.2 g/1 mL	*C. albicans* colony count	[50]
	liquid		-	
*Cymbopogon* *citratus* (lemongrass) (Poaceae)	2.5 and 5 vol.% (oil)	TC/Moonstar Soft ((Moonstar, Turkey) 10 g/7.8 mL	-	[51]
	liquid		Peel bond strength, roughness	
*Cocos nucifera* (coconut) (Arecaceae)	5, 10, 15, 20, 25, 30, 35, 40 vol./vol. % (oil extract)	TC/Visco-gel (Dentsply DeTrey, Konstanz, Germany) 3 g/2 mL	*C. albicans* inhibition zone	[45]
	liquid		-	
	1.5, 2.5 vol/vol. % oil	ST-A-SLM/Vertex Soft (Vertex Dental, Soesterberg, The Netherlands) 1.2 g/1 mL	-	[52]
	liquid		Shore A hardness	
*Allium sativum* (garlic) (Amaryllidaceae)	50, 100, 200, 400, 500 µg (powder)	TC/GC Soft Liner (GC Corp., Tokyo, Japan) 1.2 g/1 mL	*C. albicans* colony count	[44]
	powder		Shore A hardness	
	50 µg/mL (oil)	TC/Acrosoft (Marlik Medical Industries Co., Tehran, Iran) 3.2 g/2.5 mL	*C. albicans* inhibition zone	[47]
	formulation		-	
*Litsea cubeba* (Lauraceae)	5, 10, 20, 30 vol./vol. % (oil extract)	TC/GC Soft Liner (GC Corp., Tokyo, Japan) 1.2 g/1 mL TC/Visco-gel (Dentsply DeTrey, Konstanz, Germany) 3 g/2 mL TC/Coe-Comfort (COE, GC America Inc., Alsip, IL, USA) 6 g/5 mL	*C. albicans* and *S. mutans* inhibition zone	[53]
	liquid		Shore A hardness	
*Cinnamomum* (cinnamon) (Lauraceae)	1 and 2 wt. % (oil)	ST-A-SLM/Vertex Soft (Vertex Dental, Soesterberg, The Netherlands) 1.2 g/1 mL	*C. albicans* inhibition zone	[54]
	liquid		Shore A hardness	
*Curcuma longa* (curcumin) (Zingiberaceae)	10 and 20 vol./vol. % (10 vol. % ethanolic extract)	TC/Trusoft (Boswoth Company, Skokie, IL, USA) 9 g/6.8 mL	*C. albicans* inhibition zone	[55]
	liquid		Tensile bond strength	
*Zingiber officinale* (ginger) (Zingiberaceae)	50 µg/mL (oil)	TC/Acrosoft (Marlik Medical Industries Co., Tehran, Iran) 3.2 g/2.5 mL	*C. albicans* inhibition zone	[47]
	formulation		-	
*Nigella sativa* (black seed) (Ranunculaceae)	50 µg/mL (oil)	TC/Acrosoft (Marlik Medical Industries Co., Tehran, Iran) 3.2 g/2.5 mL	*C. albicans* inhibition zone	[47]
	formulation		-	
*Piper bettle* (Piperaceae)	2.5, 5, 10, 20, 30, 40 wt./wt. % (crude extract) and 2.5, 5, 10, 20, 30, 40 vol./vol. % (oil extract)	TC/GC Soft Liner (GC Corp., Tokyo, Japan) 1.2 g/1 mL	*C. albicans* and *S. mutans* inhibition zone	[34]
	formulation		-	
	0.25, 0.5, 1, 2.5, 5, 10, 20 wt./wt. % (oil extract)	TC/GC Soft Liner (GC Corp., Tokyo, Japan) 1.2 g/1 mL	*C. albicans* inhibition zone	[56]
	liquid		Shore A hardness	
*Sesamum indicum* (sesame) (Pedaliaceae)	5 vol.% (oil)	ST-A-SLM/Vertex Soft (Vertex Dental, Soesterberg, The Netherlands) 1.2 g/1 mL	-	[48]
	liquid		Tensile strength	
Chitosan	2 wt. % (powder)	TC/GC Soft Liner (GC Corp., Tokyo, Japan) 1.2 g/1 mL	*C. albicans* colony count	[40]
	powder		-	
	1.5 and 2 wt. % (powder/nanoparticles)	ST-A-SLM/Vertex Soft (Vertex Dental, Soesterberg, The Netherlands) 1.2 g/1 mL	*C. albicans* colony count	[57]
	liquid		-	
	0.625, 1.25 and 2.5 mg/mL (powder)	TC/GC Soft Liner (GC Corp., Tokyo, Japan) 1.2 g/1 mL	*C. albicans* colony count	[58]
	powder		-	

**Table 2:** Comparative overview of silicone-based SLMs (LT-SLMs) modified via addition method.

Plant Latin Name (Common Name) (Family)	Additive Content (Form)	SLM Type/Name (Manufacturer, Country) Base/Catalyst Volume Ratio	Antimicrobial Properties	Reference
	Enriched SLM Component		Mechanical Properties	
*Origanum vulgare* (Carvacrol) (Lamiaceae)	10 µL/5 mm disc (essential oil)	LT-S-SLM/Ufi Gel SC (UG, VOCO GmbH, Cuxhaven, Germany) 1:1	*C. albicans*, *S. aureus*, *S. sanguis*, *S. mutans, B. subtilis*, *P. aeruginosa*, and *E. coli* inhibition zone	[59]
	formulation		-	
*Melaleuca* *alternifolia* (tea tree) (Myrtaceae)	15 wt. % (oil)	LT-S-SLM/GC Reline Extra Soft (GC Dental Industrial Corp., Tokyo, Japan) 1:1	*C. albicans* colony count	[60]
	formulation		-	

**Table 3:** Comparative overview of acrylic-based SLMs (TCs) modified via the immersion method.

Plant Latin Name (Common Name) (Family)	Immersion Liquid (Volume)	SLM Type/Name (Manufacturer, Country) Powder/Liquid Ratio	Antimicrobial Properties	Reference
			Mechanical Properties	
*Azadirachta indica* (Neem) (Meliaceae)	alcoholic extract (10 mL)	TC/GC Soft Liner (GC Corp., Tokyo, Japan) 1.2 g/1 mL	*C. albicans* colony count	[61]
			-	
	10 wt.% alcoholic extract (10 mL)	TC/GC Soft Liner (GC Corp., Tokyo, Japan) 1.2 g/1 mL	*C. albicans*, *S. mutans* and *E. coli* colony count	[62]
			-	
	oil (10 mL)	TC/GC Soft Liner (GC Corp., Tokyo, Japan) 1.2 g/1 mL	*C. albicans* colony count	[61]
			-	
*Melaleuca* *alternifolia* (tea tree) (Myrtaceae)	10 vol. % extract (10 mL)	TC/GC Soft Liner (GC Corp., Tokyo, Japan) 1.2 g/1 mL	*C. albicans*, * S. mutans* and *E. coli* colony count	[62]
			-	
	50 vol. % DMSO extract (not specified volume)	TC/GC Soft Liner (GC Corp., Tokyo, Japan) 1.2 g/1 mL	*C. albicans* biofilm formation	[63]
			Shore A hardness	
*Cymbopogon* *citratus* (lemongrass) (Poaceae)	50 vol. % DMSO extract (aqueous extract)	TC/GC Soft Liner (GC Corp., Tokyo, Japan) 1.2 g/1 mL	*C. albicans* biofilm formation	[63]
			Shore A hardness	
*Thymus vulgaris* (thyme) (Lamiaceae)	50 vol. % DMSO extract (not specified volume)	TC/GC Soft Liner (GC Corp., Tokyo, Japan) 1.2 g/1 mL	*C. albicans* biofilm formation	[63]
			Shore A hardness	

**Table 4:** Comparison of in vitro antifungal assays against *C. albicans* of SLMs modified with plant-derived additives (IZD—inhibition zone diameter, CFU—colony forming unit, MIC—minimum inhibitory concentration, TC—tissue conditioner, ST-A-SLM—short term acrylic soft lining material, and LT-S-SLM—long term silicone soft lining material).

Plant Latin Name (Common Name) Family	SLM Type Name (Polymer/Plasticizer)	Results	Reference
*Litsea cubeba* (Lauraceae)	TCs: Visco-gel (PEMA/triethyl citrate) Coe-Comfort (PEMA)/benzyl benzoate) GC Soft Liner (PMMA/butyl phthalate butyl glycolate)	IZD for 10, 20, and 30 vol./vol.%: Visco-gel: 12.09, 16.56, 24.68 mm Coe-Comfort: 10.22, 14.07, 22.61 mm GC Soft Liner: 7.61, 13.19, 22.42 mm Antifungal efficacy: Visco-gel > Coe-Comfort > GC Soft Liner	[53]
*Melaleuca* *alternifolia* (tea tree) (Myrtaceae)	TCs: Visco-gel (PEMA/triethyl citrate) GC Soft Liner (PMMA/butyl phthalate butyl glycolate)	IZD for 10 vol./vol.%: Visco-gel: 19.0–20.8 mm GC Soft Liner: 0–17.4 mm IZD for 40 vol./vol.%: Visco-gel: 21.6–26.4 mm GC Soft Liner: 15.2–23.2 mm Fungal reduction (for 2 mL, 40 vol./vol.%): Visco-gel 88% GC Soft Liner 86% Antifungal efficacy: Visco-gel > GC Soft Liner	[50]
*Melaleuca* *alternifolia* (tea tree) (Myrtaceae)	LT-S-SLM GC Reline Extra Soft (Vinyl dimethyl polysiloxane/hydrogen polysiloxane)	Log CFU/mm^2^ for 1, 30, 60 days (15 wt.%): 2.1, 2.8, 3.1 Fungal reduction: 70.4%, 56.9%, 54.4% Sustained antifungal activity up to 60 days	[60]
*Cymbopogon* *citratus* (lemongrass) (Poaceae)	TC GC Soft Liner (PMMA/butyl phthalate butyl glycolate)	MICs/Fungal reduction: *C. citratus*: 0.03125%/50% *T. vulgare*: 0.0625%/30% *M. aternifolia*: 0.25%/20% Antifungal activity: *C. citratus* > *T. vulgare* > *M. alternifolia*	[63]
*Thymus vulgaris* (thyme) (Lamiaceae)			
*Melaleuca* *alternifolia* (tea tree) (Myrtaceae)			
*Azachirachta* *indica* (Neem) (Meliaceae)	TC GC Soft Liner (PMMA/butyl phthalate butyl glycolate)	Log CFU/mL: *M. alternifolia*: 2.31 *A. indica*: 2.15 Antifungal activity: *A. indica* > *M.alternifolia*	[62]
*Melaleuca* *alternifolia* (tea tree) (Myrtaceae)			
*Melaleuca* *alternifolia* (tea tree) (Myrtaceae)	TC GC Soft Liner (PMMA/butyl phthalate butyl glycolate)	Log CFU/mL: *M. alternifolia*: 2.30 *M. alternifolia* + *A. indica*: 0.40 Antifungal activity: *M. alternifolia* + *A. indica* > *A. indica*	[61]
*Azachirachta* *indica* (Neem) (Meliaceae)			
*Azachirachta* *indica* (Neem) (Meliaceae)	TC Visco-gel (PEMA/triethyl citrate)	IZDs for 5–40 vol./vol.% at 48 h: *A. indica*: 6.35–20.10 mm *C. nucifera*: 5.0–16.55 mm *M. alternifolia*: 5.0–15.35 mm IZDs for 40 vol./vol.% at 7 days: *A. indica*: 19.15 mm *C. nucifera*: 16.20 mm *M. alternifolia*: 14.75 mm Optimal concentrations: *A. indica*: 15 vol./vol.% *C. nucifera*: 20 vol./vol.% *M. alternifolia*: 25 vol./vol.% Antifungal activity: *A. indica* > *C. nucifera* > *M. alternifolia*	[45]
*Cocos nucifera* (coconut) (Arecaceae)			
*Melaleuca* * alternifolia* (tea tree) (Myrtaceae)			
*Azachirachta* * indica* (Neem) (Meliaceae)	TC GC Soft Liner (PMMA/butyl phthalate butyl glycolate)	100% fungal reduction: *A. indica*: 7 days *A. sativum*: 2 days Antifungal activity: *A. indica* > *A. sativum*	[44]
*Allium sativum* (garlic) (Amaryllidaceae)			
*Allium sativum* (garlic) (Amaryllidaceae)	TC Acrosoft (PEMA/phthalate ester)	IZD: *A. sativum*: 8.00 mm *O. vulgare*: 7.75 mm *Z. officinale* and *N. sativa*: negligible Antifungal activity: *A. sativum* > *O. vulgare* > *Z. officinale* ≈ *N. sativa*	[47]
*Origanum vulgare* (oregano) (Lamiaceae)			
*Zingiber officinale* (ginger) (Zingiberaceae)			
*Nigella sativa* (black seed) (Ranunculaceae)			
*Origanum vulgare* (oregano) (Lamiaceae) (carvacrol)	LT-S-SLM Ufi Gel SC (Mixture of different polyalkylsiloxanes)	IZD: 38.33 mm Fungal reduction: 98%	[59]
*Centratherum* *anthelminticum* (bitter cumin) (Asteraceae)	TC Visco-gel (PEMA/triethyl citrate)	IZD: *C. anthelminticum* (600–800 µL): 31.66–18.33 mm (7 days) *O. sanctum* (800 µL): 21.00–29.66 mm (7 days) L. *usitatissimum* (800–1000 µL): 20.33–21.00 mm (24 h) Antifungal activity: *C. anthelminticum* > *O. sanctum > L. usitatissimum*	[46]
*Ocimum sanctum* (Holy Basil and Tulsi) (Lamiaceae)			
*Linum* *usitatissimum* (flaxseed) (Linaceae)			
*Piper bettle* (Piperaceae)	TC GC Soft Liner (PMMA/butyl phthalate butyl glycolate)	IZD: 40 wt./wt.% extract: 26.63 mm 40 vol./vol.% oil: 13.69 mm Antifungal activity: extract > essential oil	[34]
*Ocimum basilicum* (basil) (Lamiaceae)	TC Visco-gel (PEMA/triethyl citrate)	MICs/Fungal reduction after 24 h and 14 days: 0.0625 mL/60.03% and 65.94% 0.25 mL/55.06% and 52.68	[49]
*Curcuma longa* (curcumin) (Zingiberaceae)	TC Trusoft (PEMA/benzyl butyl phthalate, dibutyl phthalate)	IZD: 10 vol./vol.%: 6 mm 20 vol./vol.%: 8.2 mm	[55]
*Cinnamomum* (cinnamon) (Lauraceae)	ST-A-SLM Vertex Soft (PEMA/acetyl tributyl citrate)	IZD: 1 wt.%: 12.56 mm 2 wt.% 16.72 mm	[54]
*Aloe vera* (aloe) (Liliaceae)	TC GC Soft Liner (PMMA/butyl phthalate butyl glycolate)	Fungal reduction: 80%	[40]
Chitosan	ST-A-SLM Vertex Soft (PEMA/acetyl tributyl citrate)	Fungal reduction: 1.5 wt.%: 50% 2 wt.%: 70%	[57]
Chitosan	TC GC Soft Liner (PMMA/butyl phthalate butyl glycolate)	Fungal reduction: 2 wt.%: 50%	[40]
Chitosan (commercial)	TC GC Soft Liner (PMMA/butyl phthalate butyl glycolate)	MIC: Chitosan commercial: 0.625 mg/mL Chitosan synthesized: 0.3125 mg/mL Antifungal activity: chitosan synthesized > chitosan commercial	[58]
Chitosan (synthesized)			

**Table 5:** Comparison of in vivo antifungal activity of SLM (non-specified) modified with plant-derived fungicides.

Plant Latine Name (Common Name) Family	Methodology	Results	Reference
*Azadirachta indica* (Neem) (Meliaceae)	5 mg/mL (equivalent to 2 tablets) aqueous solution (overnight immersion)	Fungal reduction: *A. Indica*: 71% Triphala: 51% *A. vera*: 41% Antifungal activity: *A. Indica* > Triphala > *A. vera*	[77]
Triphala (Combretaceae)	10 g/100 mL aqueous solution (overnight immersion)		
*Aloe vera* (aloe) (Liliaceae)	Leaves (rubbing the denture)		

**Table 6:** Comparison of in vitro antibacterial assays of SLMs modified with plant-derived additives (IZD—inhibition zone diameter, CFU—colony forming unit, TC—tissue conditioner).

Plant Latine Name (Common Name) Family	SLM Type Name (Polymer/Plasticizer)	Results/Findings	Reference
*Azadirachta indica* (Neem) (Meliaceae)	TC GC Soft Liner (PMMA/butyl phthalate butyl glycolate)	Log CFU/mL: *S. mutans*: 2.14 *S. aureus*: 2.44 *E. coli*: 6.22 Antibacterial activity: *S. mutans* > *S. aureus* > *E. coli*	[61]
*Piper betle* (Piperaceae)	TC GC Soft Liner (PMMA/butyl phthalate butyl glycolate)	IZD (*S. mutans*): 10–70 wt./wt.% extract: 8.38–26.76 mm 60–70 vol./vol.% oil: 8.31–9.30 mm Antibacterial activity: extract (70 wt./wt.%) > oil (70 vol./vol.%)	[34]
*Melaleuca alternifolia* (tea tree) (Myrtaceae)	TC GC Soft Liner (PMMA/butyl phthalate butyl glycolate)	Log CFU/mL: *S. aureus:* 2.21 *S. mutans:* 2.33 *E. coli:* 2.45 Antibacterial activity: *S. aureus > S. mutans > E. coli*	[62]
*Litsea cubeba* (Lauraceae)	TCs: Coe-Comfort (PEMA)/benzyl benzoate) Visco-gel (PEMA/triethyl citrate) GC Soft Liner (PMMA/butyl phthalate butyl glycolate)	IZD (*S. mutans*): Coe-Comfort: 8.15 mm Visco-gel: 7.96 mm GC Soft Liner: 7.89 mm Antibacterial activity: Coe-Comfort > Visco-gel > GC Soft Liner	[53]
*Origanum vulgare* (Carvacrol) (Lamiaceae)	LT-S-SLM Ufi Gel SC (Mixture of different polyalkylsiloxanes)	IZD/Bacteria reduction: *B. subtilis:* 43.67 mm/90% *S. mutans:* 40.33 mm/99% *S. sanguis:* 36.67 mm/96% *S. aureus:* 34.00 mm/97% *E. coli*: 29.33 mm/70% *P. aeruginosa:* 15.33 mm/68% Antibacterial activity: *B. subtilis* > *S. mutans* > *S. sanguis* > *S. aureus* > *E. coli* > *P. aeruginosa*	[59]

**Table 7:** LD_50_ values of plant-derived antimicrobials, administered orally in mice and rats.

Plant	Preparation Way	Test Species	LD_50_ (mg/kg)	Reference
*Aloe vera* (aloe)	dried leaf extract	Swiss albino mice	120.65	[78]
	aqueous extract of leaves	female Wistar rats	>5000	[79]
*Azachirachta indica* (Neem)	seed oil	rats	>14,000	[80]
		mice	2500	[81]
			>5000	[82]
	methanolic extract of leaves	albino rats	>5000	[83]
	methanolic extract of flowers	Wistar rats	>12,000	[84]
	ethanolic extract of leaves	mice	>5000	[85]
	ethanolic extracts of the stem bark	rats	870	[86]
		mice	489.90	[87]
Triphala	aqueous extract	Sprague-Dawley rats	>5000	[88]
		mice	280	[89]
*Linum usitatissimum* (flaxseed)	crude oil	mice (Balb C57)	37 240	[90]
*Centratherum anthelminticum* (black cumin)	oil	Wistar albino rats	>2000	[91]
*Origanum vulgare* (oregano)	essential oil	rats	>2000	[92]
	aqueous extract		2000	[93]
*Ocimum sanctum* (Holy Basil and Tulsi)	essential oil	mice	4.571	[94]
	aqueous extract		6200	[95]
	ethanolic extract		>2000	[96]
*Ocimum basilicum* (basil)	essential oil	Swiss mice	532	[97]
		Wistar rats	3250	[98]
*Melaleuca alternifolia* (tea tree)	essential oil	rats	1900	[99]
*Cocos nucifera* (coconut)	oil	rats	>5000	[100]
		mice	>5000	[101]
*Allium sativum* (garlic)	aqueous extract	mice	>5000	[102]
			650	[103]
		rats	>30	[104]
	ethanol extract	mice	4.472	[105]
*Cymbopogon citratus* (lemongrass)	essential oil	mice	3500	[106]
		albino rats	>5000	[107]
*Thymus vulgaris* (thyme)	essential oil	rats	1220	[108]
			4700	[109]
	ethanolic extract	mice	4220	[110]
*Curcuma longa* (curcumin)	ethanol extract of leaves	Swiss albino mice	2154.06	[111]
	aqueous, methanolic and n-hexane extracts of roots	albino rats	>5000	[112]
	essential oil of rhizomes	mice	2154	[113]
	ethanolic extract of rhizomes	rats	3807	[114]
		mice	27,980	[115]
*Litsea cubeba*	essential oil	mice	4000	[116]
		rats	>5000	[117]
*Nigella sativa* (black seed)	oil	mice	>28,000	[118]
	(2-Isopropyl-5-methyl-1, 4-benzoquinone) (a bioactive compound in *N. sativa*)	rats	794.3	[119]
		mice	870.9	
*Zingiber officinale* (ginger)	ethanolic extract of the root	Wistar rats	3800	[120]
	methanolic extract of the root	mice	10,250	[121]
*Cinnamomum* (cinnamon)	aqueous extract of bark	Wistar rats	5000	[122]
	essential oil	rats	2323	[123]
		mice	1850	[124]
*Piper betle*	methanolic extract	mice	>5000	[125]
Chitosan	powder	rats	>5000	[126]

**Table 8:** LD_50_ values of common drugs and antiseptics used to treat candidiasis, administered orally in mice and rats.

Medicine	Test Species	LD_50_ (mg/kg)	Reference
nystatin	mice	200	[127]
	rats	10,000	[128]
fluconazole	rats	1575	[129]
	mice	1408	[130]
itraconazole	rats	>320	[131]
	mice	>320	
ketoconazole	rats	166	[132]
	mice	618	
miconazole	mice	578.1	[133]
	rats	>640	
clotrimazole	rats	708	[134]
	mice	761	
chlorhexidine diacetate	rats	1180	[135]
	mice	2000	
chlorhexidine digluconate	rats	2000	[136]

**Table 9:** Comparison of mechanical examinations of SLMs modified with plant-derived additives.

Plant Latine Name (Common Name) Family	SLM Type Name (Polymer/Plasticizer)	Results/Findings	Reference
*Melaleuca alternifolia* (tea tree) (Myrtaceae)	TC GC Soft Liner (PMMA/butyl phthalate butyl glycolate)	Shore A hardness after 60 days: *M. alternifolia*: 52.6 °Sh *C. citratus:* 57.1 °Sh *T. vulgaris:* 58.9 °Sh Shore A hardness: *M. alternifolia* < *C. citratus* < *T. vulgaris*	[63]
*Cymbopogon* *citratus* (lemongrass) (Poaceae)			
*Thymus vulgaris* (thyme) (Lamiaceae)			
*Allium sativum* (garlic) (Amaryllidaceae)	TC GC Soft Liner (PMMA/butyl phthalate butyl glycolate)	Shore A hardness: *A. sativum:* 13.0 °Sh *A. indica:* 14.1 °Sh Shore A hardness: *A. sativum < A. indica*	[44]
*Azachirachta indica* (Neem) (Meliaceae)			
*Piper bettle* (Piperaceae)	TC GC Soft Liner (PMMA/butyl phthalate butyl glycolate)	Shore A hardness after 2 h/7 days: 5 vol.vol.%: 19 °Sh/27 °Sh 10 vol.vol.%: 13 °Sh/23 °Sh 20 vol.vol%: 7 °Sh/15 °Sh Shore A hardness: 20 vol./vol.% < 10 vol./vol.% < 5 vol./vol.%	[56]
*Litsea cubeba* (Lauraceae)	TCs: Coe-Comfort (PEMA)/benzyl benzoate) GC Soft Liner (PMMA/butyl phthalate butyl glycolate) Visco-gel (PEMA/triethyl citrate)	Shore A hardness at 5, 10, 20, and 30 vol./vol.% after 2 h: Coe-Comfort: 13.96, 10.00, 5.20, 2.12 °Sh GC Soft Liner: 14.72, 11.24, 6.88, 4.08 °Sh Visco-gel: 34.67, 32.73, 29.07, 22.27 °Sh Shore A hardness at 5, 10, 20, and 30 vol./vol.% after 7d: Coe-Comfort: 13.96, 10.00, 5.20, 2.12 °Sh GC Soft Liner: 22.36, 18.52, 13.64, 8.84 °Sh Visco-gel: 37.93, 35.87, 32.53, 27.93 °Sh Shore A hardness: Coe-Comfort < GC Soft Liner < Visco-gel	[53]
*Cocos nucifera* (coconut) (Arecaceae)	ST-A-SLM Vertex Soft (PEMA/acetyl tributyl citrate)	Shore A hardness after 2 h/14 days/4 weeks: 1.5%: 39.63 °Sh/45.41 °Sh/42.99 °Sh 2.5%: 50.13 °Sh/55.11 °Sh/52.74 °Sh Shore A hardness: 1.5% < 2.5%	[52]
*Cinnamomum* (cinnamon) (Lauraceae)	ST-A-SLM Vertex Soft (PEMA/acetyl tributyl citrate)	Shore A hardness: 1 wt.%: 45.23 °Sh 2 wt.%: 47.13 °Sh Shore A hardness: 1 wt.% *<* 2 wt.%	[54]
*Sesamum indicum* (sesame) (Pedaliaceae)	ST-A-SLM Vertex Soft (PEMA/acetyl tributyl citrate)	Tensile Strength after 2 days/30 days: *S. indicum*: 3 MPa/3 MPa *T.vulgaris*: 3.75 MPa/3 MPa *S. indicum + T.vulgaris*: 3.60 MPa/3.20 MPa Tensile strength: *S. indicum < T.vulgaris < S. indicum + T.vulgaris*	[48]
*Thymus vulgaris* (thyme) (Lamiaceae)			
*Aloe vera* (aloe) (Liliaceae)	ST-A-SLM Vertex Soft (PEMA/acetyl tributyl citrate)	Tear strength after 24 h/2 weeks/4 weeks: 3 wt.%: 7.093 N/mm/7.92 N/mm/9.2 N/mm 10 wt.%: 5.2 N/mm/5.7 N/mm/6.45 N/mm Tear strength: 10 wt.% < 3 wt.%	[41]
*Aloe vera* (aloe) (Liliaceae)	ST-A-SLM Vertex Soft (PEMA/acetyl tributyl citrate)	Tear strength: *A. indica*: 6.7 N/mm *A. vera:* 7.4 N/mm Tear strength: *A. indica* < *A. vera*	[42]
*Azachirachta indica* (Neem) (Meliaceae)			

**Table 10:** Comparison of interface mechanical properties of SLMs modified with plant-derived additives.

Plant Latine Name (Common Name) Family	SLM Type Name (Polymer/Plasticizer)	Results/Findings	Reference
*Cymbopogon* *citratus* (lemongrass) (Poaceae)	TC Moonstar Soft (PEMA/dibutyl phthalate)	Peel bond strength: 2.5 vol./vol.%: 2.04 MPa 5 vol./vol.%: 1.83 MPa Peel bond strength: 5 vol./vol.% < 2.5 vol./vol.%	[51]
*Aloe vera* (aloe) (Liliaceae)	TC GC Soft Liner (PMMA/butyl phthalate butyl glycolate)	Shear bond strength: *A. vera:* 0.48 MPa *A. indica:* 0.56 MPa Shear bond strength: *A. vera < A. indica*	[43]
*Azachirachta indica* (Neem) (Meliaceae)			
*Aloe vera* (aloe) (Liliaceae)	ST-A-SLM Vertex Soft (PEMA/acetyl tributyl citrate)	Shear bond strength after 24 h/2 weeks/4 weeks: 3 wt.%: 0.302 MPa/0.345 MPa/0.404 MPa 10 wt.%: 0.288 MPa/0.334 MPa/0.391 MPa Shear bond strength: 10 wt.% < 3 wt.%	[41]
*Cinnamomum* (cinnamon) (Lauraceae)	TC Trusoft (PEMA/benzyl butyl phthalate, dibutyl phthalate)	Tensile bond strength: 10 vol./vol.%: 0.52 MPa 20 vol./vol.%: 0.55 MPa Tensile bond strength: 10 vol./vol.% < 20 vol./vol.%	[55]

**Table 11:** Comparison of studies on surface roughness of SLMs modified with plant-derived additives.

Plant Latine Name (Common Name) Family	SLM Type Name (Polymer/Plasticizer)	Results/Findings	Reference
*Centratherum anthelminticum* (bitter cumin) (Asteraceae)	TC Visco-gel (PEMA/triethyl citrate)	Surface roughness (SEM observations): *O. sanctum* < *C. anthelminticum* < *L. usitatissimum*	[46]
*Ocimum sanctum* (Holy Basil and Tulsi) (Lamiaceae)			
*Ocimum basilicum* (basil) (Lamiaceae)	TC Visco-gel (PEMA/triethyl citrate)	Surface roughness: 1.25 vol.%: 3.2564 µm 5 vol.%: 3.3448 µm Surface roughness: 1.25 vol.% < 5 vol.%	[49]
*Cymbopogon* *citratus* (lemongrass) (Poaceae)	TC Moonstar Soft (PEMA/dibutyl phthalate)	Surface roughness: 2.5 vol./vol.%: 2.503 µm 5 vol./vol.%: 2.352 µm Surface roughness: 5 vol./vol.% < 2.5 vol.vol.%	[51]
*Cinnamomum* (cinnamon) (Lauraceae)	TC Trusoft (PEMA/benzyl butyl phthalate, dibutyl phthalate)	Surface roughness: 10 vol./vol.%: 3.3 µm 20 vol./vol.%: 2.53 µm Surface roughness: 20 vol./vol.% < 10 vol.vol.%	[55]

## Data Availability

No new data were created or analyzed in this study. Data sharing is not applicable to this article.

## Supplementary Materials

The following supporting information can be downloaded at: https://www.mdpi.com/article/10.3390/ijms262210848/s1.

## Abbreviations

The following abbreviations are used in this manuscript:

A-SLM	Acrylic-based Soft Lining Material
BPO	Benzoyl Peroxide
BuMA	n-Buthyl Methacrylate
CFU	Colony Forming Unit
CFU/mL	Colony Forming Unit per milliliter
DD	Degree of Deacetylation
DMSO	Dimethyl Sulfoxide
DMPT	N,N-Dimethyl-*p*-toluidine
DNA	Deoxyribonucleic Acid
EGDMA	Ethylene Glycol Dimethacrylate
EMA	Poly(ethyl methacrylate)
HWP1	Hyphal Wall Protein 1
ISO	International Organization for Standardization
IZD	Inhibition Zone Diameter
LD_50_	Lethal Dose 50%
LT-SLM	Long-term Soft Lining Material
LT-S-SLM	Long-term Silicone Soft Lining Material
MAP	Mitogen-Activated Protein
MFC	Minimum Fungicidal Concentration
MIC	Minimum Inhibitory Concentration
MMA	Methyl Methacrylate
mRNA	Messenger Ribonucleic Acid
MTT	3-(4,5-dimethylthiazol-2-yl)-2,5-diphenyltetrazolium bromide
MW	Molecular Weight
PEMA	Poly(methyl methacrylate)
PRISMA	Preferred Reporting Items for Systematic Reviews and Meta-Analyses
SLM	Soft Lining Material
S-SLM	Silicone-based Soft Lining Material
ST-SLM	Short-term Soft Lining Material
T-SLM	Temporary Soft Lining Material
TC	Tissue Conditioner
TEGDMA	Triethylene Glycol Dimethacrylate
WCA	Water Contact Angle
WHO	World Health Organization
